# Intrinsic charge polarization governs hydrogen-bonding strength in amide *vs.* imide self-association

**DOI:** 10.1039/d6cp02391f

**Published:** 2026-09-03

**Authors:** Siebe Lekanne Deprez, Cécile M. M. de Jager, Eveline H. Tiekink, Stephanie C. C. van der Lubbe, Célia Fonseca Guerra

**Affiliations:** a Department of Chemistry and Pharmaceutical Sciences, AIMMS, Vrije Universiteit Amsterdam De Boelelaan 1108 Amsterdam 1081 HZ The Netherlands c.fonsecaguerra@vu.nl

## Abstract

The weaker self-association of imide dimers relative to amide dimers is often attributed to repulsive secondary electrostatic interactions (SEI) between diagonally positioned carbonyl groups. Quantification of the SEI interactions using quantum chemical analyses reveals that they are negligible and do not explain the stronger self-association of amides. Instead, amides exhibit a larger intrinsic charge polarization, resulting in a more favorable alignment of hydrogen-bond donor and acceptor regions. This enhanced polarization strengthens the electrostatic attraction between the monomers and simultaneously reinforces the covalent component of the hydrogen bond through more favorable donor–acceptor orbital interactions. Our findings demonstrate that intrinsic charge polarization within the monomers, rather than secondary electrostatic interactions between frontier atoms, governs the stability of hydrogen-bonded amide and imide dimers, highlighting the importance of quantum-mechanical descriptions for understanding hydrogen-bond strengths.

## Abstract 2

Direct quantification reveals that secondary electrostatic interactions contribute little to amide and imide dimerization. The decisive factor is intrinsic charge polarization within the monomers.

## Graphical abstract

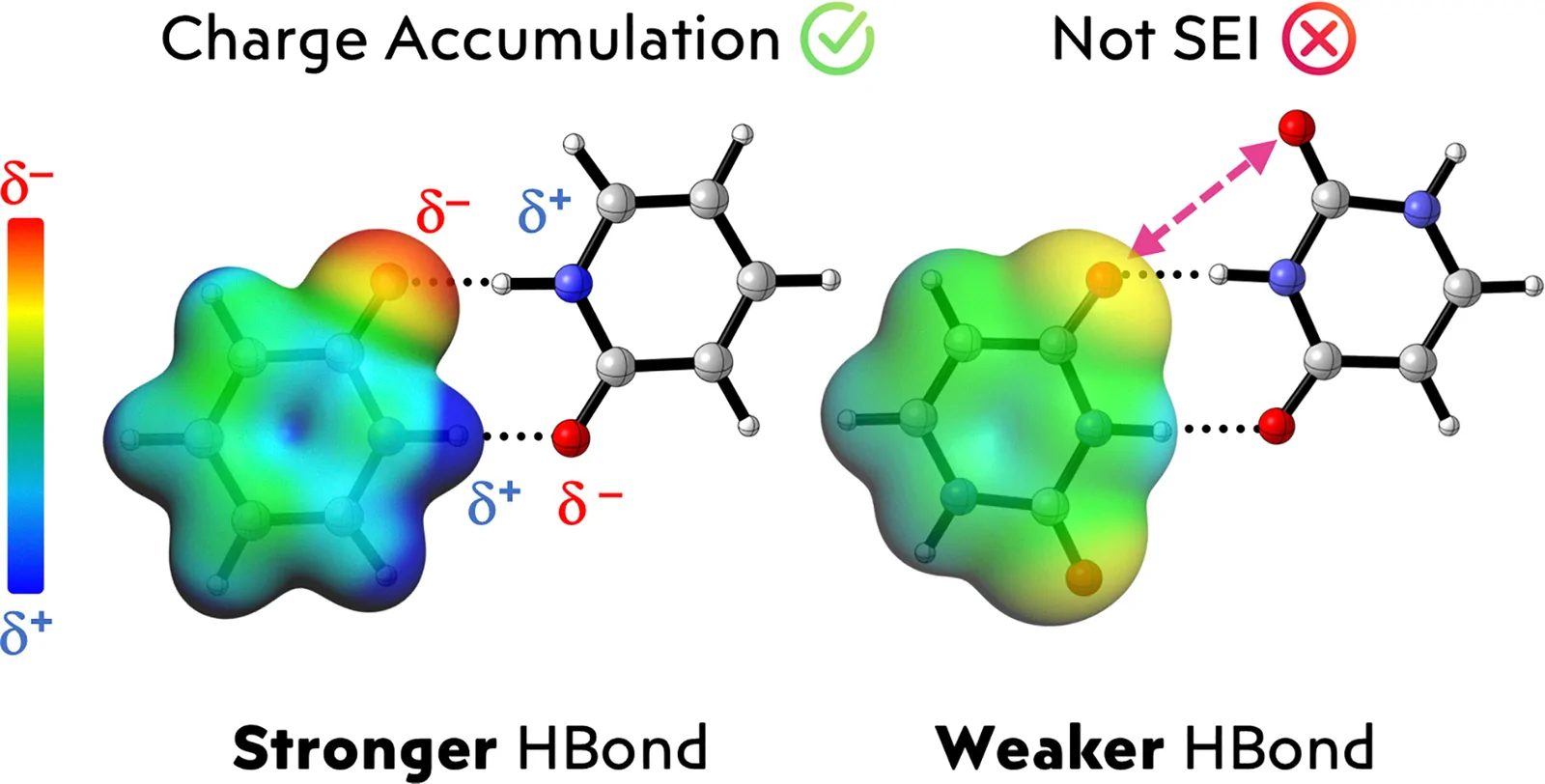

## 1 Introduction

Hydrogen bonding is a fundamental noncovalent interaction that underpins molecular recognition, supramolecular self-assembly, and the structure and function of biological systems such as proteins and nucleic acids.^1–3^ By incorporating hydrogen bonding, one enables fine-tuning material properties due to their reversibility and directionality.^4–6^ The strength of hydrogen bonding in, for example, a hydrogen-bonded dimer, depends on both the number and spatial arrangement of hydrogen-bond donors (D) and acceptors (A).

**Fig. 1:**
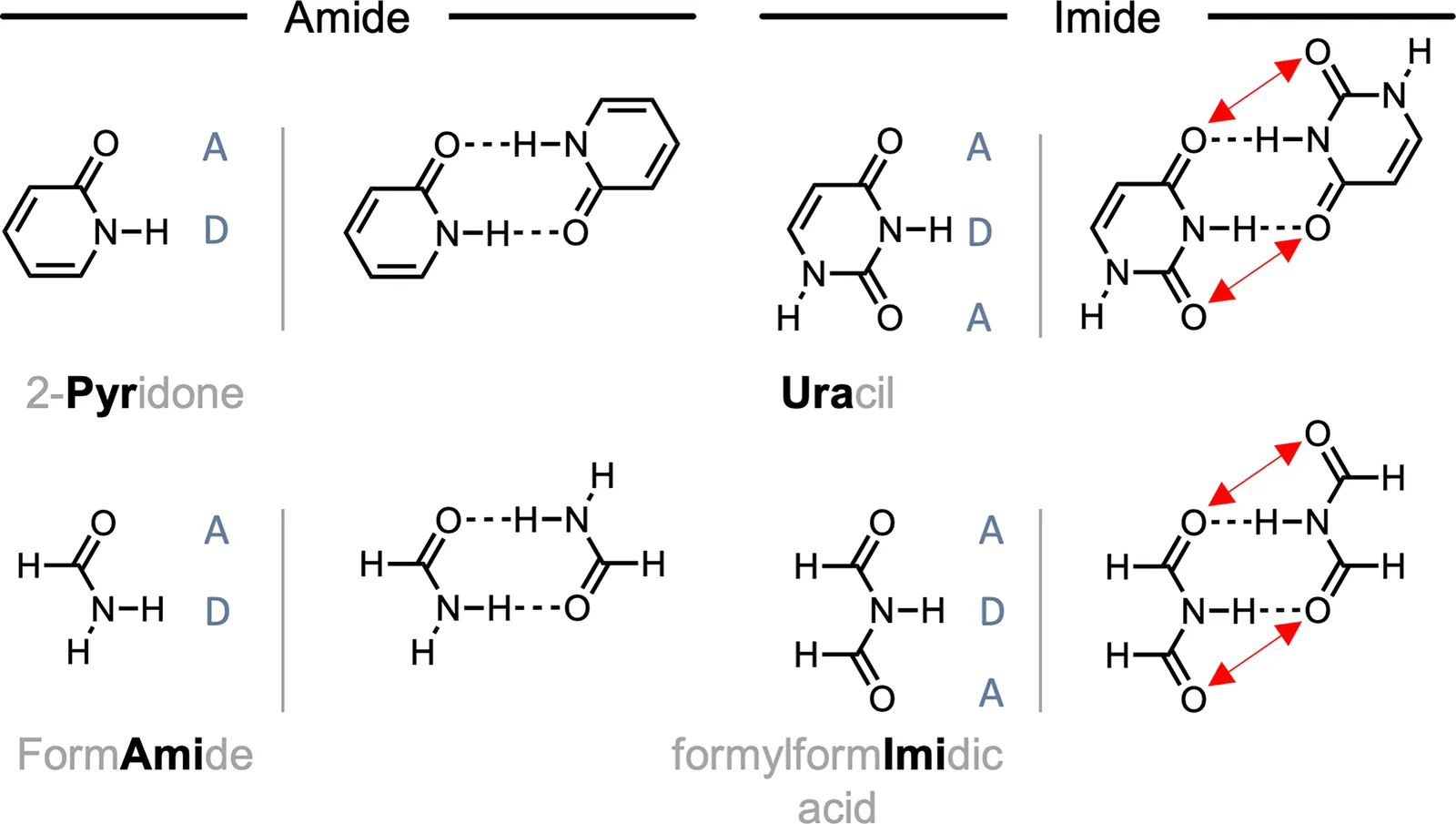
Amides and imides that are selected in this study. Hydrogen-bond donors (D) and acceptors (A) arrangements are shown. Red arrows highlight the secondary interactions that are repulsive according to the SEI model which are only present in imides and not in amides.

Previously, Rebek *et al.* observed that double hydrogen-bonded *cis*-amide dimers are more stable than structurally related imide dimers.^7,8^ Yet, imides are expected to form stronger hydrogen bonds as imide monomers possess a more acidic N–H group (see Fig. 1).^9^ This counterintuitive trend has traditionally been explained using the Secondary Electrostatic Interaction (SEI) model, originally introduced to rationalize stability differences in complexes bonded through a multifold of hydrogen bonds.^10–12^ Within this framework, the donor–acceptor sequence is key: *cis*-amides adopt an AD motif, whereas imides feature an ADA arrangement (see Fig. 1). The additional carbonyl group in imides is proposed to introduce repulsive secondary electrostatic interactions between diagonally opposed acceptors, thereby destabilizing the dimer despite identical primary hydrogen bonds.

Despite its intuitive appeal and predictive use, the SEI model has been increasingly questioned for oversimplifying the nature of hydrogen bonding.^9,13–16^ Alternative explanations have emphasized the balance between N–H acidity and C=O basicity, formalized in the acidity–basicity interplay (ABI) model, which shows that increased acidity does not necessarily translate into stronger hydrogen bonding if the acceptor basicity is simultaneously reduced.^9^ Other studies have highlighted the importance of induction effects and π-resonance, demonstrating that resonance can either enhance or weaken hydrogen bonding depending on how charge redistribution and conjugation are modulated.^14,15^ More recently, charge-density analyses have shown that hydrogen-bond strength correlates more strongly with overall charge accumulation and polarization across the monomer than with localized “secondary” interactions alone, suggesting that a more comprehensive, quantum-mechanical description is required.^13,17^

This computational work aims to investigate the origin of the stability difference between double hydrogen-bonded amide and imide complexes (see Fig. 1) by utilizing density functional theory (DFT) in the framework of Kohn–Sham molecular orbital theory (KS-MO^18,19^). We found that the amide dimer is indeed more stable than the imide dimer. The secondary electrostatic interactions model is further examined and quantified through performing Energy Decomposition Analyses (EDA^20,21^) and Voronoi Deformation Density analyses (VDD^22,23^). The “repulsive secondary electrostatic interaction” between the separated carbonyl groups is found to be very weak, almost negligible. Instead, we propose that the higher degree of intrinsic charge separation within the amide monomer explains why amides form stronger hydrogen bonds than imides.

## 2 Results and discussion

### 2.1 Bond energies and lengths

**Fig. 2:**
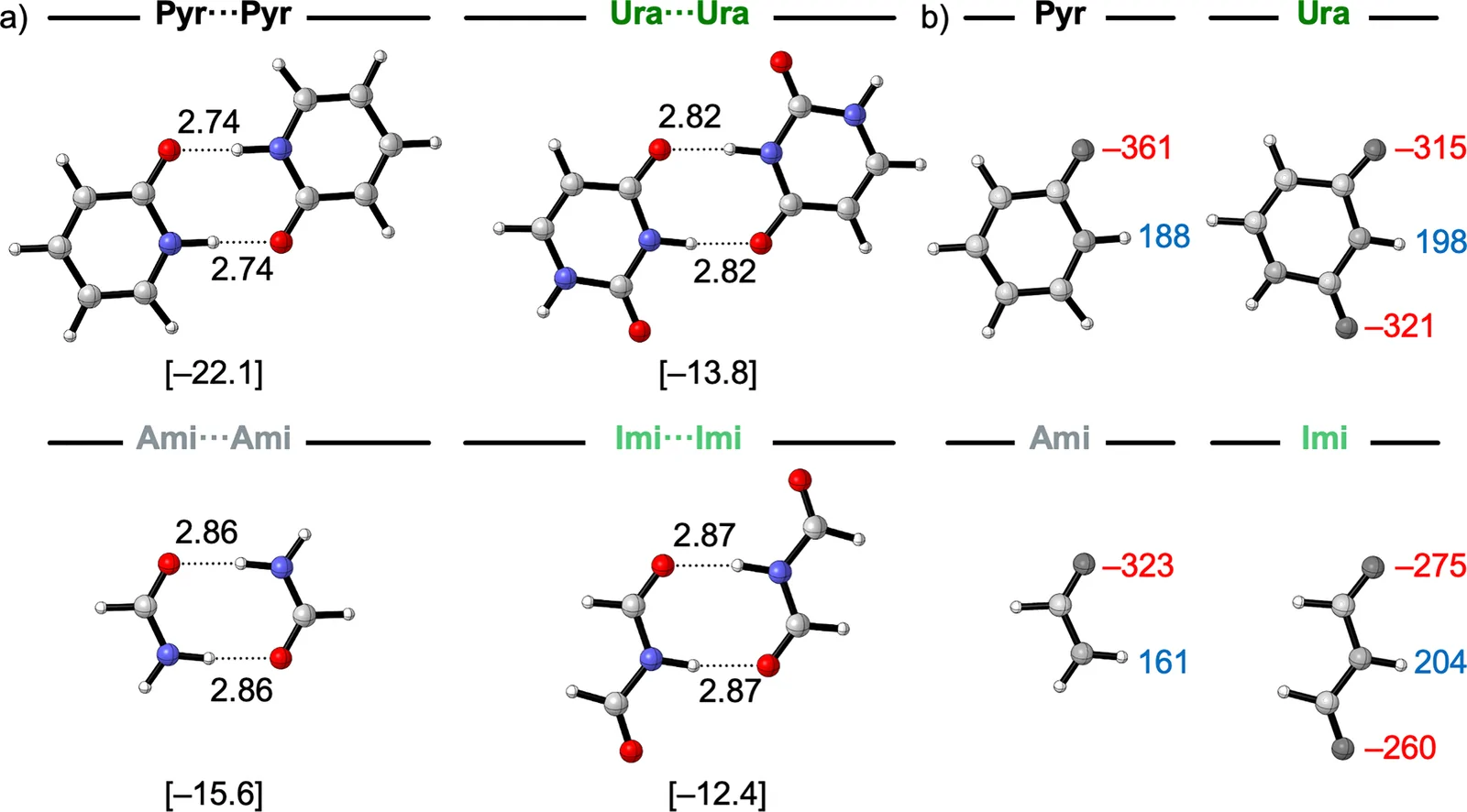
(a) Optimized dimers (adopting *C*_2h_ symmetry) with the hydrogen-bond energy (in kcal mol^−1^ between square brackets) and their hydrogen bond distances *r*_O⋯(H)N_ (in Å). (b) VDD charges (in millielectrons) of frontier atoms in the optimized monomers (adopting *C*_s_ symmetry).

We selected exemplary *cis*-amides and imides that are relevant moieties in biomolecular systems, including nucleic acids. These include amide 2-pyridone (Pyr) and imide uracil (Ura) with the latter one having incorporated an additional nitrogen and carbonyl group. To study the hydrogen-bonding strengths, dimers were created and optimized, resulting in planar, hydrogen-bonded complexes (see Fig. 2a and Table S1 in the SI). We observe that hydrogen bonding in [Pyr]_2_ is stronger than in [Ura]_2_ with stabilization energies of −22.1 kcal mol^−1^ and −13.8 kcal mol^−1^, respectively. The stronger hydrogen bonds correlate with a decrease in hydrogen-bond lengths since [Pyr]_2_ has a bond distance, *r*_O⋯(H)N_, of 2.74 Å, while the same bond is 2.82 Å in [Ura]_2_. Our findings are in line with earlier studies regarding trends in hydrogen-bond strengths for various amide and imides.^8,9,14,16,24^ In particular, Vallejo Narváez, Jiménez, and co-workers report dimerization constants of various amides and imides based on NMR experiments and DFT calculations.^9^ Their results show that [Pyr]_2_ has a dimerization constant of 460.0 M^−1^, whereas the corresponding values for imides range from 1 to 4 M^−1^. These experimental findings are consistent with our results, supporting the conclusion that amides form stronger hydrogen-bonded dimers than imides.

Furthermore, atomic charges of the frontier atoms were computed using the Voronoi deformation density (VDD) scheme (see Fig. 2b). In Pyr, the carbonyl oxygen atom is more negatively charged (−361 millielectrons in Pyr*vs.* −315 millielectrons in Ura) while the corresponding N–H hydrogen atom is less positively charged (188 millielectrons in Pyr*vs.* 198 millielectrons in Ura). Thus, the atomic charges of the frontier atoms do not directly explain the difference in hydrogen-bond strength. Furthermore, there is no quantification possible of the SEIs with the atomic charges of the additional carbonyl oxygen of Ura which is assumed to be a repulsive interaction (see Fig. 1).

Next, we want to isolate hydrogen bonding interactions by eliminating ring conjugation effects in the monomer as all atoms in the ring are sp^2^-hybridized. We simplified Pyr to formamide (Ami) and Ura to formylformimidic acid (Imi) and calculated the dimerization energies with respect to the lowest conformer (see Fig. 2a and Fig. S1 in the SI for the relative stabilities of Imi conformers). Removing the ring does not influence the trend in hydrogen bonding.^25^ [Ami]_2_ remains stronger bound than [Imi]_2_ by 3.2 kcal mol^−1^ and the *r*_O⋯(H)N_ is shorter by 0.01 Å for [Ami]_2_ than for [Imi]_2_ although less pronounced. The simplified complexes thus exhibit the same trends in hydrogen-bond energy and distance, justifying the simplification. Yet, also for Ami and Imi, the atomic charges of the frontier atoms do not explain the difference in hydrogen bond strength and the ambiguity regarding the role of an additional carbonyl oxygen in Imi remains (*i.e.*, more SEI repulsion or weaker primary hydrogen bonds).

### 2.2 Energy decomposition analysis

**Scheme 1:**
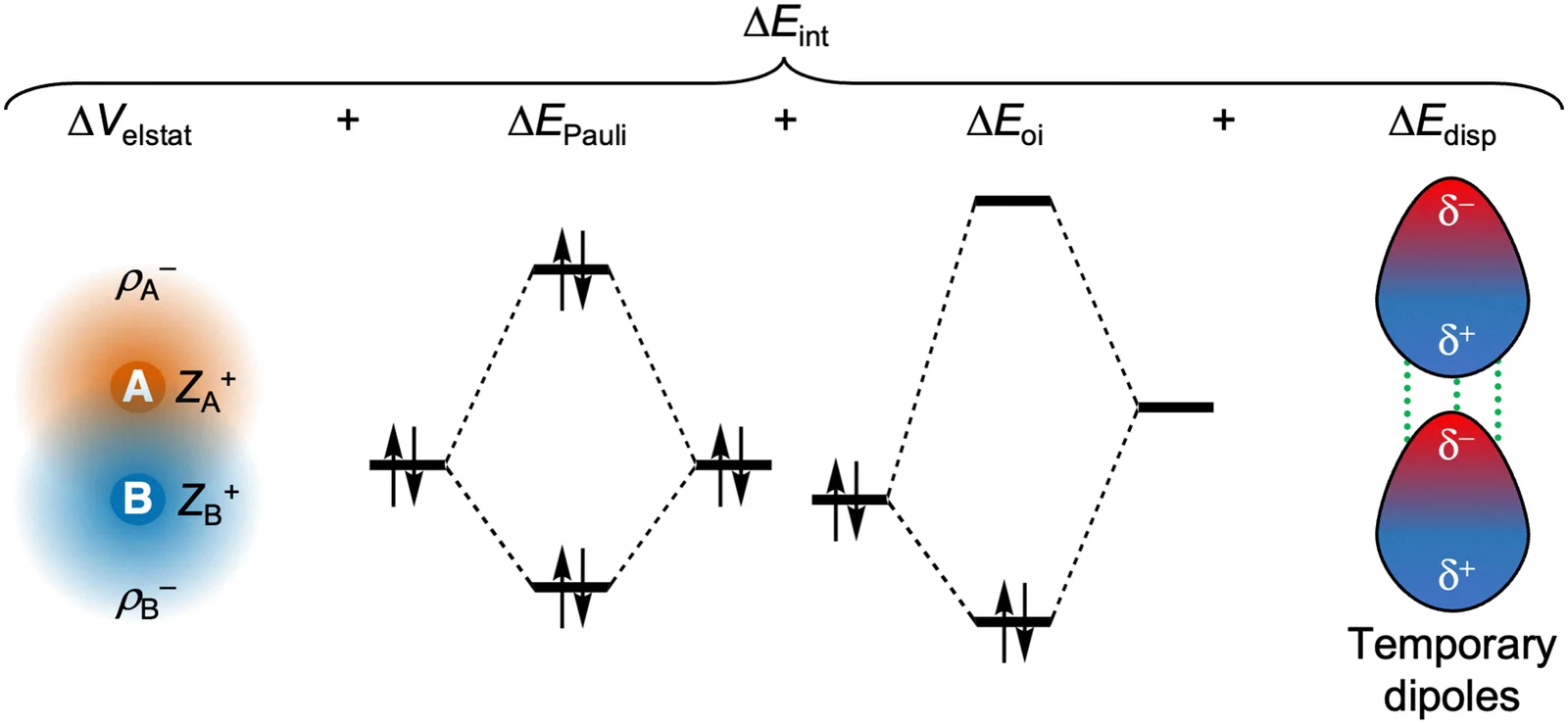
Schematic representation of the energy decomposition analysis (EDA) that decomposes the interaction energy, Δ*E*_int_, into four physically meaningful terms. *Z*^+^ and *ρ*^−^ represent the nuclei and electron density of fragments associated with fragment A (*Z*_A_^+^ and *ρ*_A_^−^) or fragment B (Z_B_^+^ and *ρ*_B_^−^).

In this section, we want to trace the importance of the electrostatic interactions in the hydrogen bonding of the four dimers. According to the SEI model, the reason why [Pyr]_2_ and [Ami]_2_ are stronger bound than [Ura]_2_ and [Imi]_2_ is because additional repulsive secondary interactions are present in the latter between the C=O moieties (see Fig. 1).^11^ As shown in previous studies,^9,13–15,17^ factors other than SEIs may play a determining role in the stronger self-association of the [Pyr]_2_ dimers, and the simplified [Ami]_2_ dimer. Herein, we perform energy decomposition analyses (EDA) to decompose the hydrogen-bond energy into physically meaningful interaction components (see Scheme 1 and eqn (3) in the Theoretical Methods for a more elaborate description).

The first term, Δ*V*_elstat_, envelops the electrostatic contribution that originates from the interaction between (unperturbed) charge densities of the monomers; the second term, Δ*E*_Pauli_, is the Pauli repulsion which arises from the overlap between occupied orbitals on the interacting monomers and constitutes the physical origin of steric repulsion; the third term, Δ*E*_oi_, is the orbital interactions stemming from polarization and charge-transfer in and between the monomers; and the last term, Δ*E*_disp_, is a dispersion correction that is small for these systems (see Section S2 in the SI).

**Fig. 3:**
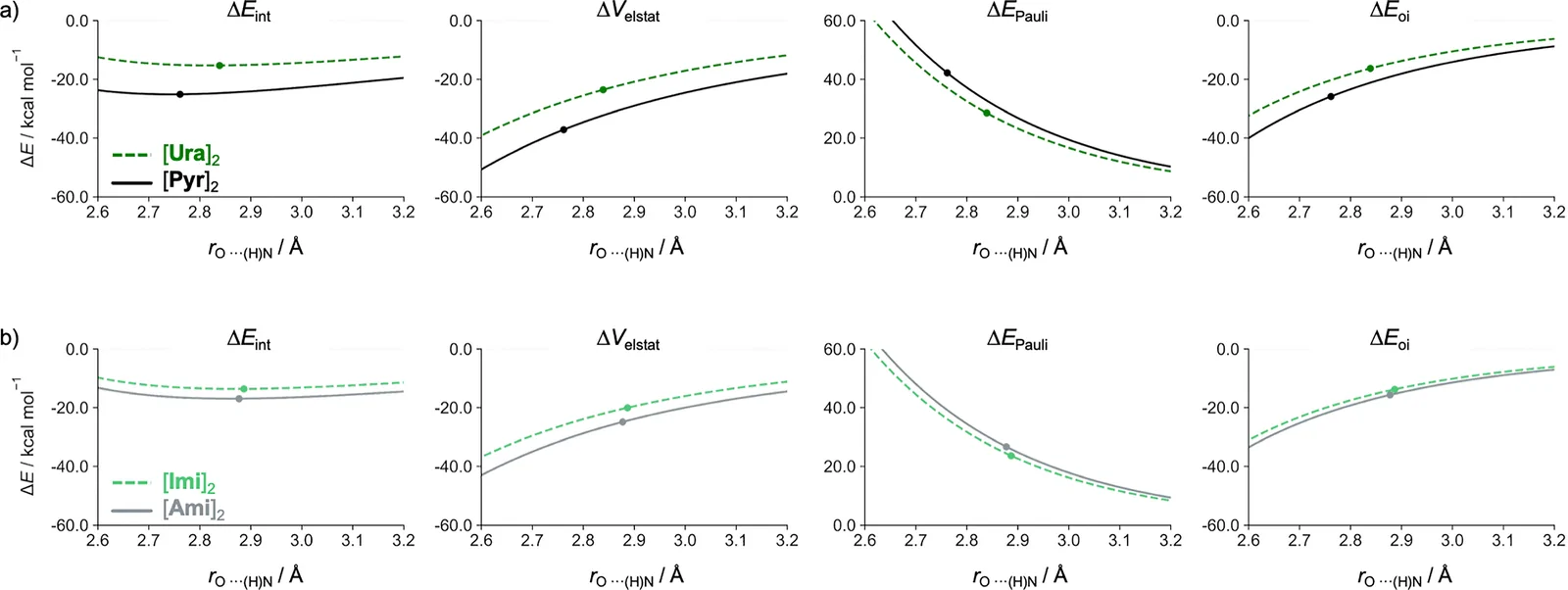
Energy decomposition analysis terms (in kcal mol^−1^) as a function of the hydrogen bond distance *r*_O⋯(H)N_ (in Å) for (a), the hydrogen-bonded complexes [Pyr]_2_ and [Ura]_2_, and (b), for the simplified analogues [Ami]_2_ and [Imi]_2_. The dots indicate the equilibrium *r*_O⋯(H)N_ distance.

Since the equilibrium *r*_O⋯(H)N_ distances vary by a maximum of 0.08 Å (see Fig. 2), which may obscure hydrogen bond effects (*i.e.*, every interaction component weakens at longer distances with respect to the equilibrium distance), we computed the EDA over an *r*_O⋯(H)N_ interval from 2.6 Å to 3.2 Å (see Fig. 3), a procedure which was previously established.^26,27^ Along this distance, monomers were frozen in the geometry they adopt in the fully optimized complex to isolate geometric deformations from the hydrogen bond interaction itself. The plots show that the interaction energy, Δ*E*_int_, of [Pyr]_2_ is significantly more stabilizing than [Ura]_2_ at every distance. At a common reference geometry of *r*_O⋯(H)N_ = 2.78 Å (*i.e.*, the average of the equilibrium *r*_O⋯(H)N_ distances in [Pyr]_2_ and [Ura]_2_), Δ*E*_int_ is 9.8 kcal mol^−1^ more stabilizing for [Pyr]_2_ which agrees with the trend in hydrogen-bond energy Δ*E* (see Fig. 2a). A similar analysis considering the simplified structures [Ami]_2_ and [Imi]_2_ reveals the same trend in Δ*E*_int_ since the grey, solid line associated with [Ami]_2_ lies above green, dashed line that belongs to [Imi]_2_ (see Fig. 2b), although the energy differences are less pronounced at both the equilibrium and common reference geometries (see Table S2 in the SI for numerical values).

The reason why Δ*E*_int_ is more stabilizing for the amide dimers is primarily because of stronger electrostatics Δ*V*_elstat_ (Fig. 3). We observe that Δ*V*_elstat_ shows a similar trend in Δ*E*_int_ as both the solid curves of [Pyr]_2_ and [Ami]_2_ lie below the dashed curves of [Ura]_2_ and [Imi]_2_, meaning the [Pyr]_2_ and [Ami]_2_ experience more stabilizing electrostatic interactions than their respective [Ura]_2_ and [Imi]_2_ at all *r*_O⋯(H)N_ distances. In contrast, the curves belonging to Δ*E*_Pauli_ show a reversed order in which the solid lines lie above the dashed lines, indicating that [Pyr]_2_ and [Ami]_2_ experience more repulsion with respect to their counterpart [Ura]_2_ or [Imi]_2_. Therefore, the trend in Δ*E*_Pauli_ goes against the trend in Δ*E*_int_ and is thus not the determining term. Furthermore, Δ*E*_oi_ also shows a similar trend compared to Δ*V*_elstat_ since solid curves lie below the dashed curves. However, the curves of Δ*E*_oi_ lie much closer together than the curves belonging to Δ*V*_elstat_ and hence, Δ*V*_elstat_ term contributes more than the Δ*E*_oi_ term to Δ*E*_int_.

Thus, the electrostatic component of hydrogen bonds is decisive in explaining why *cis*-amides form stronger hydrogen bonds than imides, which appears to be in agreement with the Secondary Electrostatic Interaction model.^10,11^ Yet, as we will show below in the framework of Kohn–Sham molecular orbital theory, amide dimers are more stable because of better alignment of charge distributions within the respective monomers, not because of less repulsive secondary interactions.

### 2.3 Electrostatic interactions

**Fig. 4:**
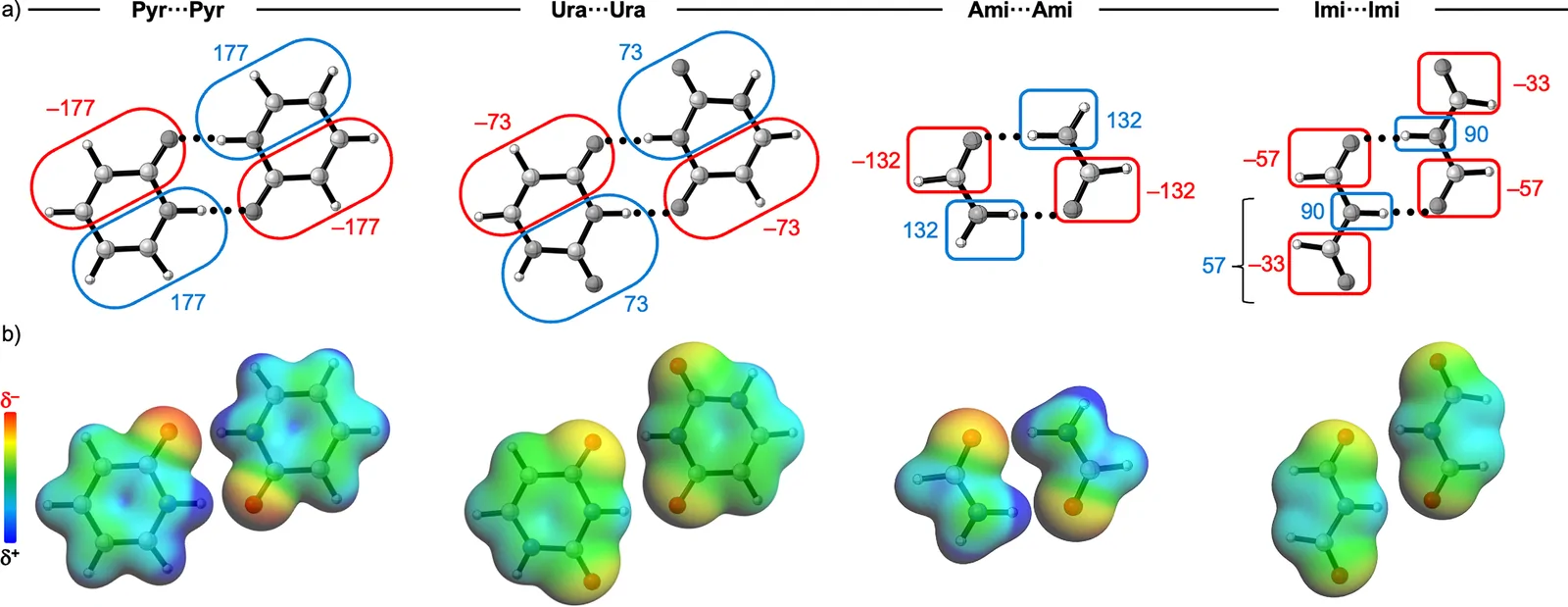
(a) VDD charges (in millielectrons) of the isolated monomers in the geometry of optimized [Pyr]_2_, [Ura]_2_, [Ami]_2_, and [Imi]_2_ dimers. Values in red indicate negative charges while in blue positive charges. (b) Molecular electrostatic potential (MEP) surfaces of the isolated monomers considering the density isosurface with an isovalue of 0.01 a.u. and with electrostatic potential values ranging from −0.1 (red) to 0.1 (blue) a.u.

The electrostatic interactions were further examined by computing the Voronoi deformation densities (VDD) atomic charges of Pyr, Ura, Ami, and Imi (see Theoretical methods). By considering the monomers in the geometry they adopt in the complex, we can study how charge distributions of the separate monomers will interact upon hydrogen bonding, thus describing the electrostatic interactions.^11,15^ We partition the monomers into hydrogen-bond acceptor region(s) (A) and the hydrogen-bond donor region (D), and sum up the atomic VDD charges belonging to the atoms in each region (see Fig. 4a). For Pyr and Ura, we have chosen to keep the fragments as similar as possible to perform the charge comparison on equal footing. The Pyr and Ami monomers exhibit a clear “up-down” charge polarization in which the hydrogen-bond acceptor moiety is negatively charged (−177 millielectrons for Pyr and −132 millielectrons for Ami), and hydrogen-bond donor moiety positively charged (+177 millielectrons for Pyr and +132 millielectrons for Ami). Upon forming hydrogen bonds in [Pyr]_2_ and [Ami]_2_, the charge distributions of each monomer align excellently, much better than the monomers in [Ura]_2_ and [Imi]_2_ which are less polarized (−73 and +73 millielectrons in Ura and −57 and +57 millielectrons in Imi). The molecular electrostatic potential (MEP) surfaces (see Fig. 4b) further indicate that the charge distribution in the Pyr dimer is better aligned for hydrogen bonding than in the Ura analogue: the hydrogen-bond donor region is bluer and the hydrogen-acceptor region redder compared to regions in [Ura]_2_, reflecting larger absolute MEP values at the isosurface which is consistent with more stabilizing electrostatic interactions Δ*V*_elstat_ (see Fig. 3). A similar trend is observed for [Ami]_2_ and [Imi]_2_, with the former exhibiting more pronounced (*i.e.*, higher contrast) regions and the latter showing more neutral MEP surfaces.

### 2.4 Fragmentations

In the previous section, we have been able to explain the stronger electrostatic interactions in the Pyr and Ami dimers in terms of VDD charges, and this section, we partition the electrostatic interactions through defining fragments in the monomers, allowing us to test the SEI model directly. We consider the same fragmentation scheme as in Fig. 4a defined by the round rectangles. Each fragment is created by cleaving its bonds to the remainder of the monomer and terminating the resulting dangling bonds with hydrogen atoms. Partial charges are subsequently assigned to each fragment, corresponding to those obtained in the complete monomer. For example, we partitioned 2-pyridone (Pyr) into a hydrogen-bond acceptor CH_2_=CH–CHO (prop-2-enal) and a hydrogen-bond donor CH_2_=CH–NH_2_ (aminoethylene). The acceptor fragment carries a partial charge of −0.177, meaning that we add 0.177 electrons to its π_LUMO_. Conversely, the donor fragment carries a partial charge of +0.177, and 0.177 electrons are removed from its π_HOMO_. Further details on the fragmentation protocol and charge assignment are provided in Section S3 of the SI.

**Fig. 5:**
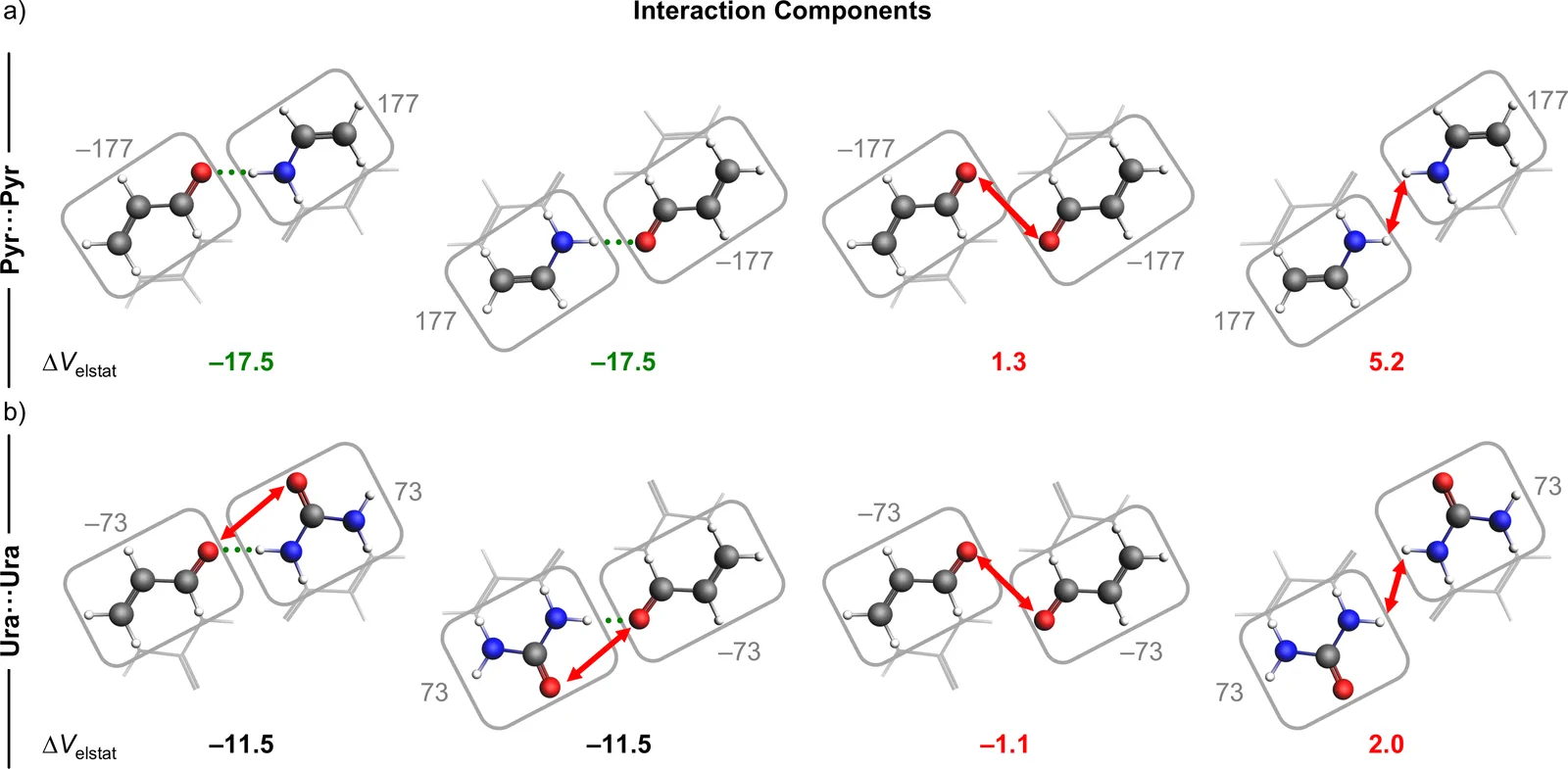
Electrostatic contributions Δ*V*_elstat_ (in kcal mol^−1^) of (a) fragmentations of the Pyr⋯Pyr interaction into primary and secondary interactions between prop-2-al and aminoethylene, and (b) fragmentations of the Ura⋯Ura interaction into primary (in green) and secondary interactions (in red) between prop-2-al and urea. Partial charges are assigned to the hydrogen-bond acceptor and donor (grey values and boxes, in millielectrons), given by the VDD charges of the full dimers (shown in Fig. 4). Calculated at a common reference geometry with a *r*_O⋯(H)N_ distance of 2.780 Å.

Now, we can compute the primary interaction (*i.e.*, hydrogen bonds) and the secondary interactions for [Pyr]_2_ at the *r*_O⋯(H)N_ distance of 2.780 Å. The results show that the two primary hydrogen bonds (in green) account for most of the interaction, being responsible for a total stabilization of –17.5 × 2 = –35.0 kcal mol^−1^ while the two repulsive SEIs (in red) amount to 1.3 kcal mol^−1^ and 5.2 kcal mol^−1^. Considering [Ura]_2_, a similar partitioning has been carried out: 0.073 electrons were added to the hydrogen-bond acceptor fragment (prop-2-enal) and 0.073 electrons were removed from the hydrogen-bond donor fragment (urea). At the same *r*_O⋯(H)N_ distance of 2.780 Å, the SEIs for [Ura]_2_ amount to respectively –1.1 kcal mol^−1^ and 2.0 kcal mol^−1^, being even slightly attractive and less repulsive than the [Pyr]_2_ dimer, in contrast to what the SEI model predicts. Furthermore, the negatively charged and positively charged fragments of Ura experience an electrostatic attraction of −11.5 kcal mol^−1^ which encompasses a primary and secondary interactions (green dotted line and red arrow in Fig. 5b). This fragmentation, which was chosen to simulate the charge separation (see Fig. 4), does not allow for the separation of the extra secondary interaction. Consequently, the hydrogen-bond donor fragment urea in Ura can be further fragmented in ammonia and formamide, and the mixed interaction of −11.5 kcal mol^−1^ can be decomposed into a primary hydrogen-bond contribution of −12.1 kcal mol^−1^ and a repulsive SEI of 0.6 kcal mol^−1^ (see Fig. S4 in the SI).

**Fig. 6:**
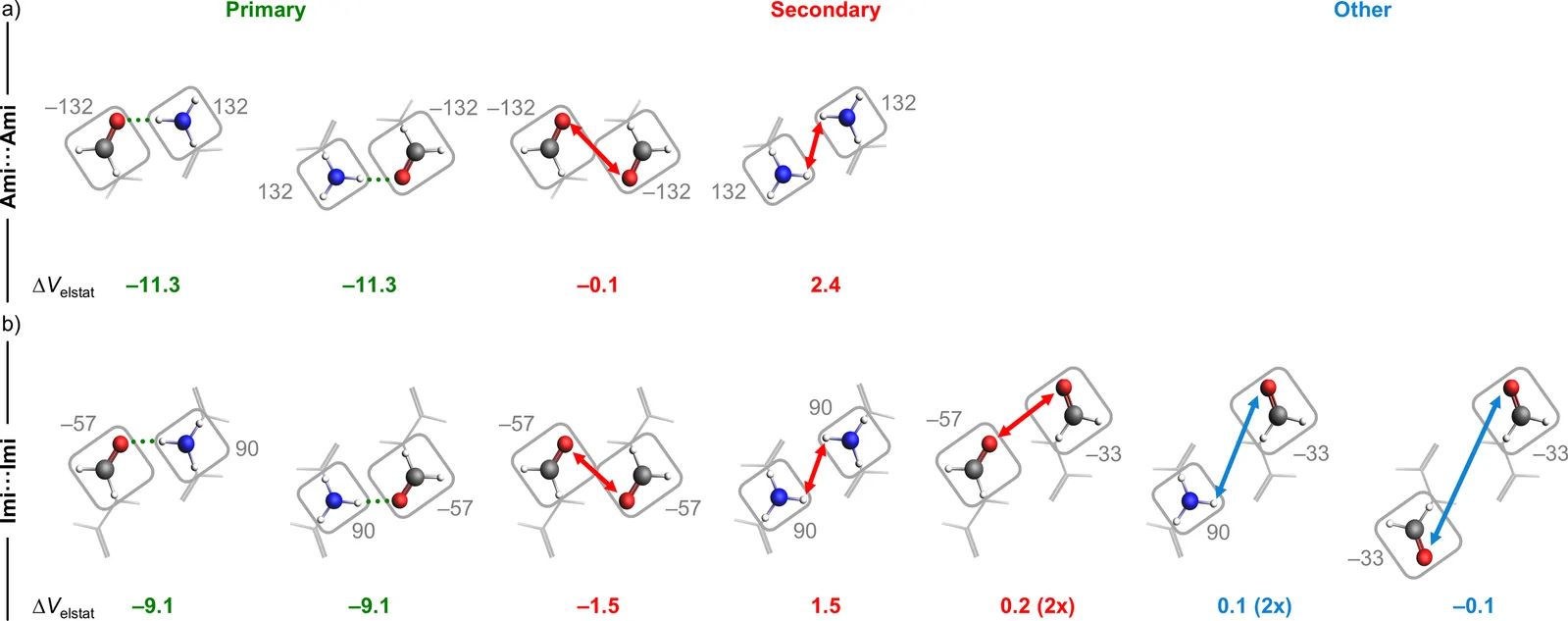
Electrostatic contributions Δ*V*_elstat_ (in kcal mol^−1^) of (a) fragmentations of the Ami⋯Ami interaction into primary and secondary interactions between formaldehyde and ammonia, and (b) fragmentations of the Imi⋯Imi interaction into primary (in green) and secondary interactions (in red). Calculated at a common reference geometry with a *r*_O⋯(H)N_ distance of 2.865 Å. Partial charges are assigned to the hydrogen-bond acceptor and donor (grey values and boxes, in millielectrons), given by the VDD charges of the full dimers (shown in Fig. 4).

The SEI model can be tested more closely through considering the simplified amide and imide dimers [Ami]_2_ and [Imi]_2_. We identify hydrogen bond donors and acceptors moieties and assign the charges as defined in Fig. 4a (see Section S3 in the SI for details about the fragmentation and assignment of charges). Our results show that the secondary interactions are almost negligible for the strength of the electrostatic component in the hydrogen bonds. Moreover, the primary interactions almost fully account for the total electrostatic interaction between the monomers. Fig. 6 shows that the primary interactions, that is, the two hydrogen bonds, are much more stabilizing than other (secondary) interactions by an order of magnitude. For instance, the primary electrostatic interactions in [Ami]_2_ are stabilizing by −22.6 kcal mol^−1^ (two times −11.3 kcal mol^−1^) while the secondary electrostatic interactions are slightly (de)stabilizing by −0.1 kcal mol^−1^ and 2.4 kcal mol^−1^ for the carbonylic interaction CH_2_O⋯CH_2_O and amidic interaction NH_3_⋯NH_3_, respectively. The primary electrostatic interactions in [Imi]_2_ are less attractive, amounting to −18.2 kcal mol^−1^ (two times −9.1 kcal mol^−1^), and the secondary electrostatic interactions are even less relevant as they sum up to only 0.5 kcal mol^−1^. Notice that one of the SEIs in [Imi]_2_ is attractive and not repulsive as suggested by the SEI model.^11^

Therefore, our analysis of the electrostatic interactions in the amide dimers compared to the imide dimers reveals that the stronger electrostatic interaction in amide dimers is mainly due to more stabilization stemming from the primary interaction between the hydrogen-bond donor and hydrogen-bond acceptor.

### 2.4 Orbital interactions

We showed in Fig. 3 that the orbital interactions in the amide dimers are stronger than in the imide dimers: at a *r*_O__⋯__(H)N_ distance of 2.780 Å, Δ*E*_oi_ in [Pyr]_2_ and [Ura]_2_ amount to −24.7 kcal mol^−1^ and −19.3 kcal mol^−1^. The smaller systems [Ami]_2_ and [Imi]_2_ are stabilized by −16.2 kcal mol^−1^ and −14.6 kcal mol^−1^ at a *r*_O__⋯__(H)N_ distance of 2.865 Å.

**Fig. 7:**
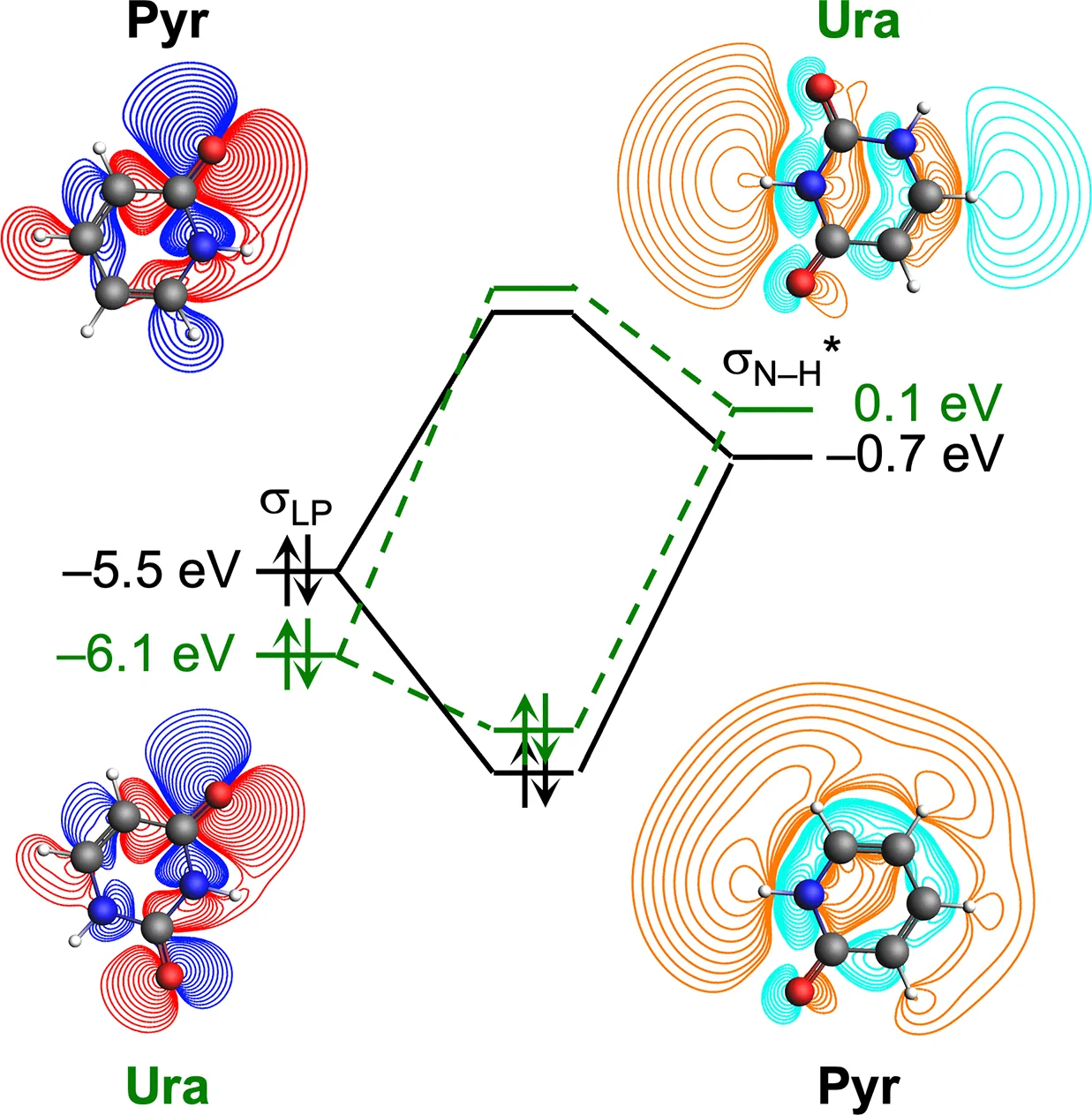
Molecular orbital diagrams for of [Pyr]_2_ and [Ura]_2_ with orbital energies *ε* (in eV), calculated at a common reference geometry with a *r*_O⋯(H)N_ distance of 2.780 Å and with planar (*C*_s_) symmetry enforced.

The difference in Δ*E*_oi_ can be explained by considering relevant orbitals in the hydrogen-bonded complex. In previous work,^24,26–29^ we showed that the most relevant orbital interactions are in the σ electronic system, that is charge-transfer interactions between the occupied σ lone pair orbitals on the hydrogen bond acceptor (oxygen or nitrogen atoms) and the unoccupied σ* anti-bonding orbitals on the hydrogen bond donor (N–H groups).^27,30^ Here, we consider the orbital interaction between the *σ*_LP_ on the HB acceptor (that is the σ_HOMO_ of Pyr and of Ura), and the *σ*_N–H_* of the HB donor (that is the *σ*_LUMO_ of Pyr and the *σ*_LUMO+1_ of Ura) which are the main orbitals responsible for the covalent component in the hydrogen bonding (see Fig. 7).

The *σ*_LP_ of Pyr lies higher in energy than the *σ*_LP_ of Ura, and the *σ*_N–H_* of Pyr lies lower in energy than the *σ*_N–H_* of Ura (see Fig. 7 and Section S4 in the SI for the full MO diagram). This difference leads to a smaller *σ* energy gap for [Pyr]_2_ (4.8 eV) than for [Ura]_2_ (6.2 eV) which strengthens the orbital interactions for the former.^31^ The same holds for the other equivalent hydrogen bond as the dimers adopt a *C*_2h_ symmetry.

The reason for the higher lying *σ*_LP_ and the lower lying *σ*_N–H_* for Pyr is because of the intrinsic polarization mentioned earlier: the *σ*_LP_ and *σ*_N–H_* are predominantly located on, respectively, the hydrogen-bond acceptor and hydrogen-bond donor regions. The acceptor region is (partially) negatively charged and the donor region (partially) positively charged. In case of the *σ*_LP_, the electrons in that orbital are repelled by the additional negative charge and thus the *σ*_LP_ is pushed up in energy. Conversely, the *σ*_N–H_* lowers in energy because the electrons experience greater attraction. This effect is more pronounced in [Pyr]_2_ than in [Ura]_2_ because of the larger charge separation within the monomers (*vide supra*).

## 3 Conclusion

Quantum-chemical analyses reveal that the stronger hydrogen bonding in amide dimers relative to imide dimers cannot be explained by the Secondary Electrostatic Interaction (SEI) model. According to this model, the weaker binding of imide dimers originates from repulsive interactions involving the additional carbonyl group. However, direct quantification of the primary hydrogen-bond interactions and the secondary electrostatic interactions demonstrates that the stability difference originates from stronger primary hydrogen bonds in the amide dimers. The contribution of secondary electrostatic interactions is small and, in some cases, even slightly stabilizing, contrary to the predictions of the SEI model.

The stronger primary hydrogen bonds in amide dimers arise from a larger intrinsic charge polarization within the amide monomers. This enhanced polarization creates a more favorable alignment of hydrogen-bond donor and acceptor regions, resulting in a stronger electrostatic attraction upon complexation. At the same time, it strengthens the covalent component of the hydrogen bond through more favorable donor–acceptor orbital interactions, reflected in a higher-lying σ lone pair on oxygen and a lower lying unoccupied antibonding σ_N–H_* orbital on the opposing N–H group.

Taken together, these findings challenge the SEI model for its oversimplified interpretation of hydrogen-bonding interactions by revealing that both polarization and orbital interactions, not secondary electrostatic interactions, are key contributors to the stronger self-assembly of amide dimers. It highlights the need for a more comprehensive model to accurately describe hydrogen bond strength in self-associating systems.

## 4 Theoretical methods

### 4.1 Computational details

All calculations were carried out using the Amsterdam Density Functional (ADF) 2025.103 module of the Amsterdam Modeling Suite.^32–35^ All stationary points and energies were calculated at the BLYP level of the generalized gradient approximation (GGA) exchange functional developed by Becke (B^36^), and the GGA correlation functional developed by Lee, Yang, and Parr (LYP^37^) (see Section S5 in the SI for the Cartesian coordinates). The DFT-D3(BJ) method, developed by Grimme and co-workers,^38^ which contains the damping function proposed by Becke and Johnson,^39^ was used to describe non-local dispersion interactions. In addition, relativistic effects were accounted for through the zeroth-order regular approximation (ZORA) method.^40^

This level of theory is referred to as ZORA-BLYP-D3(BJ)/TZ2P and has been proven to accurately describe weak interactions.^41–43^ A large uncontracted relativistically-optimized TZ2P basis set was used with no frozen-core approximation consisting of Slater type orbitals (STOs) which is of triple-ζ quality for all atoms and was augmented with the following sets of polarization functions: p and d functions on H, d and f functions on N, C, O, S and Se.^44^ Notably, previous studies on hydrogen-bonded systems have shown that the employed level of theory, *i.e.*, BLYP-D3(BJ)/TZ2P and also M06-2X, gives a basis set superposition error (BSSE) of only a few tenths of a kcal mol^−1^ and hence does not affect the computed trends in hydrogen-bond strength.^43,45^ To show the similar outcomes between BLYP-D3(BJ)/TZ2P and the M06-2X XC functional, the latter was also employed on the geometries obtained with the BLYP-D3(BJ) XC functional, yielding the ZORA-M06-2X/TZ2P//ZORA-BLYP-D3(BJ)/TZ2P level of theory.^43^ The molecular density is fitted by the systematically improvable Zlm fitting scheme and numerical integration is performed on a Becke grid.^46,47^ Both were set to “VeryGood” for geometry optimizations, and to “Excellent” for fragment analyses. Finally, the geometries were visualized using the CYLview2.0.^48^

### 4.2 Bond energy analysis

The hydrogen-bond strength, Δ*E*, of a hydrogen-bonded complex is defined as shown in eqn (1), where *E*_complex_ represents the energy of the interacting monomers, *E*_monomer1_ and *E*_monomer2_:

1 Δ*E* = *E*_complex_ − (*E*_monomer1_ + *E*_monomer2_)

To understand the different components that determine the hydrogen-bond strength, Δ*E* can be partitioned as formulated by eqn (2) according to the activation strain model (ASM).^49–53^ In this model, Δ*E* comprises two components, Δ*E*_strain_ and Δ*E*_int_. Here, the strain energy (Δ*E*_strain_) is the energy required to deform the equilibrium geometry of each monomer to the geometry it acquires when it interacts in the complex. The interaction energy (Δ*E*_int_) accounts for the stabilizing interaction between the two deformed monomers.

2 Δ*E* = Δ*E*_strain_ + Δ*E*_int_

The latter term, Δ*E*_int_, can be further decomposed in the framework of the Kohn–Sham molecular orbital model, using the quantitative canonical energy decomposition analysis (EDA^19,54^): the electrostatic interaction (Δ*V*_elstat_), Pauli repulsion (Δ*E*_Pauli_), orbital interactions (Δ*E*_oi_), and an additional term that accounts for dispersion interactions Δ*E*_disp_, as shown in eqn (3).

3 Δ*E*_int_ = Δ*V*_elast_ + Δ*E*_Pauli_ + Δ*E*_oi_ + Δ*E*_disp_

The Δ*V*_elstat_ term represents the quasi-classical Coulomb interaction between the unperturbed charge distributions of the deformed monomers. Δ*E*_Pauli_ comprises destabilizing interactions between occupied orbitals on each monomer and is responsible for steric repulsion. The orbital interaction energy (Δ*E*_oi_) accounts for donor–acceptor interactions and polarization effects, including interactions between the highest occupied and lowest unoccupied MOs (HOMO–LUMO). Finally, Δ*E*_disp_ accounts for dispersion corrections as introduced by Grimme *et al.*^38^ The ASM and EDA analyses were performed using the PyFrag 2019 program.^55,56^

### 4.3 Voronoi deformation density charge analysis

For computing atomic charges and analyzing the electron density distribution, the Voronoi deformation density (VDD) method was used.^22^ The VDD atomic charge on atom A in a molecule (*Q*^VDD^_A_) is computed as the (numerical) integral of the deformation density in the volume of the Voronoi cell of A [eqn (4)]. The Voronoi cell of A is defined as the compartment of space bounded by the bond midplanes on and perpendicular to all bond axes between nucleus A and its neighboring nuclei.4
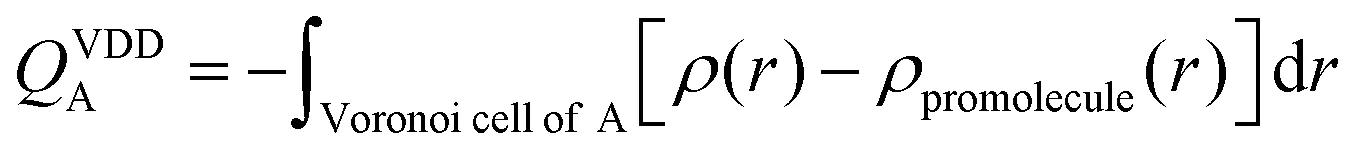
Here, the deformation density is the difference between *ρ*(*r*), *i.e.*, the electron density of the overall molecule or complex, and 
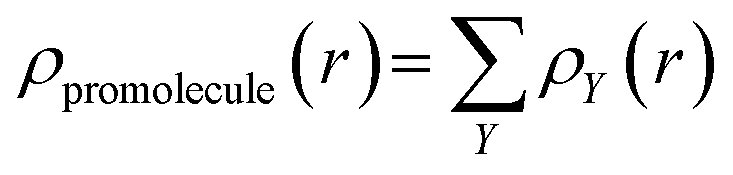
, *i.e.*, the superposition of spherical average-of-configuration atomic densities *ρ*_*Y*_(*r*) of each atom Y in the fictitious promolecule without chemical interactions, in which all atoms are considered neutral. The terms *ρ*_promolecule_(*r*) and *ρ*_*Y*_(*r*) on itself do not have a clear and distinct meaning. Instead, the deformation density, that is the difference *ρ*(*r*) − *ρ*_promolecule_(*r*), measures the charge flow to or from a nucleus A. The interpretation of the VDD charge *Q*^VDD^_A_ is now straightforward and transparent: instead of measuring the amount of charge associated with A, *Q*^VDD^_A_ directly monitors how much charge flows out of (*Q*^VDD^_A_ > 0) or into (*Q*^VDD^_A_ < 0) the Voronoi cell of A due to interactions with neighboring nuclei and electrons.

## Author contributions

S. Lekanne Deprez: methodology, validation, formal analysis, investigation, data curation, writing – original draft, visualization. C. M. M. de Jager: investigation, writing – review and editing. E. H. Tiekink: investigation, writing – review and editing. S. C. C. van der Lubbe: investigation, writing – review and editing. C. Fonseca Guerra: conceptualization, methodology, resources, supervision, writing – review and editing, project administration, funding acquisition.

## Conflicts of interest

There are no conflicts to declare.

## Supplementary Material

CP-028-D6CP02391F-s001

## Data Availability

All relevant data are either included in the main text or in the supplementary information (SI). Supplementary information: additional figures and tables, and Cartesian coordinates of the amides and imides discussed in the main text. See DOI: https://doi.org/10.1039/d6cp02391f.
