# Supplementary material for: Intrinsic charge polarization governs hydrogen-bonding strength in amide vs. imide self-association
Source: Phys Chem Chem Phys. 2026 Sep 3;28(37):22934–42. doi: 10.1039/d6cp02391f (PMC13559684; doi:10.1039/d6cp02391f)
Supplement: Supplementary file 1 [file CP-028-D6CP02391F-s001.pdf]

Supplementary Information (SI) for Physical Chemistry Chemical Physics.
This journal is © the Owner Societies 2026

Supporting Information for

Intrinsic Charge Polarization Governs Hydrogen-bonding Strength in Amide vs. Imide Self-Association

Siebe Lekanne Deprez, Cécile M. M. de Jager, Eveline H. Tiekink, Stephanie C. C. van der Lubbe, Célia Fonseca Guerra*

Department of Chemistry and Pharmaceutical Sciences, AIMMS, Vrije Universiteit Amsterdam, De Boelelaan 1108, Amsterdam 1081 HZ, The Netherlands. E-mail: c.fonsecaguerra@vu.nl.

Table of Contents

S1 Geometries 3

S2 Activation strain model and energy decomposition analysis 3

S3 Fragmentation procedure and electron occupations 5

S4 Orbital Interactions 9

S5 Cartesian coordinates 10

S1 Geometries

Table S1. Energy differences calculated at optimized geometries with (C_s/C_{2h}/C_{2h}) and without (C_1) symmetry enforced, ΔE_{sym} (in kcal mol^{-1}) for **Pyr**, **Ura**, **Ami**, and **Imi** monomers and dimers, computed at ZORA-BLYP-D3(BJ)/TZ2P. Cartesian coordinates are given in Table S4 and Table S5.

System	*Symmetry*	$\delta\Delta E_{sym}$[a]	$N_{imaginary\ frequemties}$
**Pyr**	C_1	0.0	0
	C_s	0.0	0
**[Pyr]$_2$**	C_1	0.0	0
	C_{2h}	0.0	0
**Ura**	C_1	0.0	0
	C_s	0.0	0
**[Ura]$_2$**	C_1	0.0	0
	C_{2h}	0.0	0
**Ami**	C_1	0.0	0
	C_s	0.0	0
[**Ami**]$_2$	C_1	0.0	0
	C_{2h}	0.0	0
**Imi**	C_1	0.0	0
	C_{2v}	0.0	0
[**Imi**]$_2$	C_1	0.0	0
	C_{2h}	0.0	0

[a] $\Delta E_{sym} = E_{[irrep]} - E_{C1}$

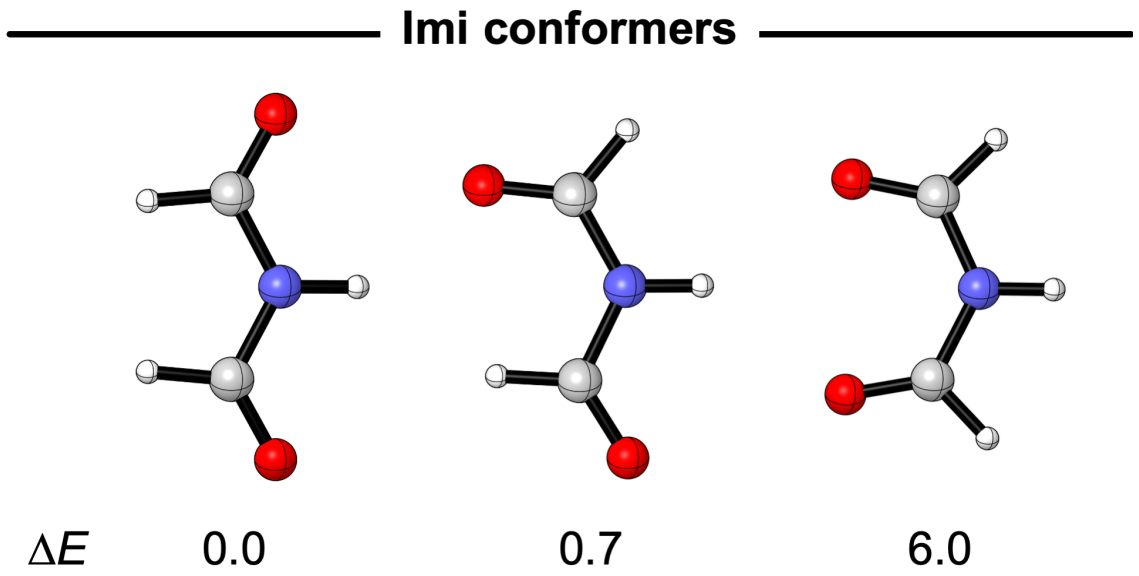

Figure S1. Relative energies of **Imi** conformers in kcal mol^{-1}, computed at ZORA-BLYP-D3(BJ)/TZ2P.

S2 Activation strain model and energy decomposition analysis

Table S2. Activation strain model (ASM) and Energy decomposition analyses (EDA) of amide dimers [Pyr]$_2$ and [Ami]$_2$, and imide dimers [**Ura**]$_2$ and [**Imi**]$_2$ at equilibrium distances and common reference geometries (or "consistent geometries", in bold). Energies values are given in kcal mol^{-1} and distances in Å.

System	$r_{O\cdots(H)N}$	ΔE	ΔE_{strain}[a]	ΔE_{int}	ΔV_{elstat}	ΔE_{Pauli}	ΔE_{oi}	ΔE_{oi}^{AA}	ΔE_{oi}^{AAA}	ΔE_{disp}
**[Pyr]$_2$**	2.744	-22.1	3.1	–25.1	–38.4	44.6	–27.1	–23.7	–3.4	–4.3
	**2.780**	-22.1		–25.1	–35.9	39.7	–24.7	–21.6	–3.1	–4.2
**[Ura]$_2$**	2.822	-13.8	1.5	–15.3	–24.4	30.2	–17.1	–15.5	–1.6	–4.0
	**2.780**	-13.7		–15.2	–26.6	34.7	–19.3	–17.4	–1.8	–4.1
[**Ami**]$_2$	2.860	-15.6	1.4	–17.0	–25.7	28.2	–16.4	–14.6	–1.8	–3.1
	**2.865**	-15.6		–17.0	–25.5	27.8	–16.2	–14.4	–1.8	–3.1
[**Imi**]$_2$	2.869	-12.4	1.2	–13.6	–20.8	25.0	–14.4	–13.0	–1.4	–3.5
	**2.865**	-12.4		–13.6	–20.9	25.4	–14.6	–13.2	–1.4	–3.5

[a] Strain energy is independent of the bond distance because the fragments were frozen in the geometry they adopt in the optimized dimer.

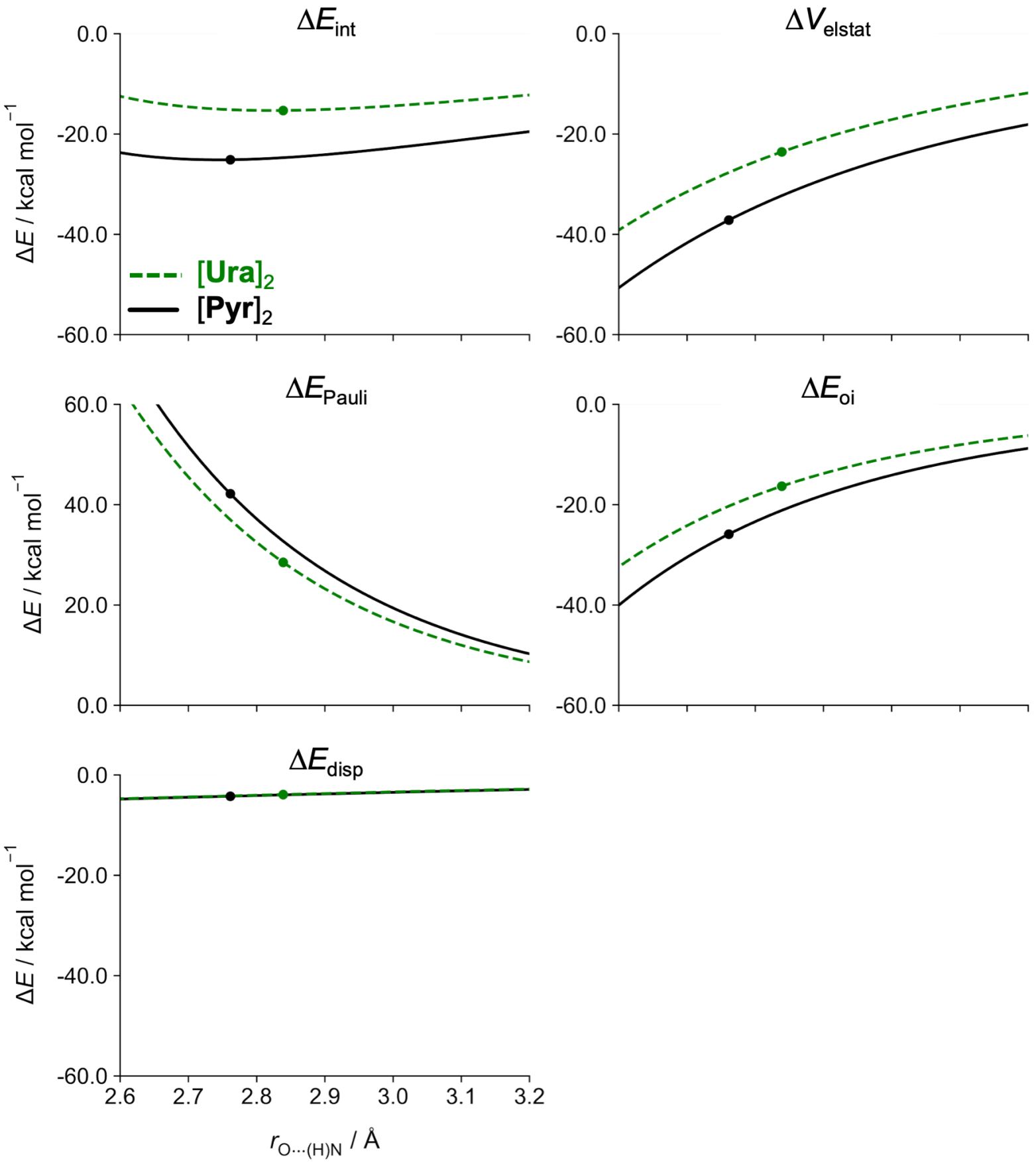

Figure S2. Energy decomposition analysis terms (in kcal mol^{-1}) including dispersion as a function of the hydrogen bond distance $r_{O\cdots(H)N}$ (in Å) for the hydrogen-bonded complexes [**Pyr**]$_2$ and [**Ura**]$_2$. The dots indicate the equilibrium $r_{O\cdots(H)N}$ distances.

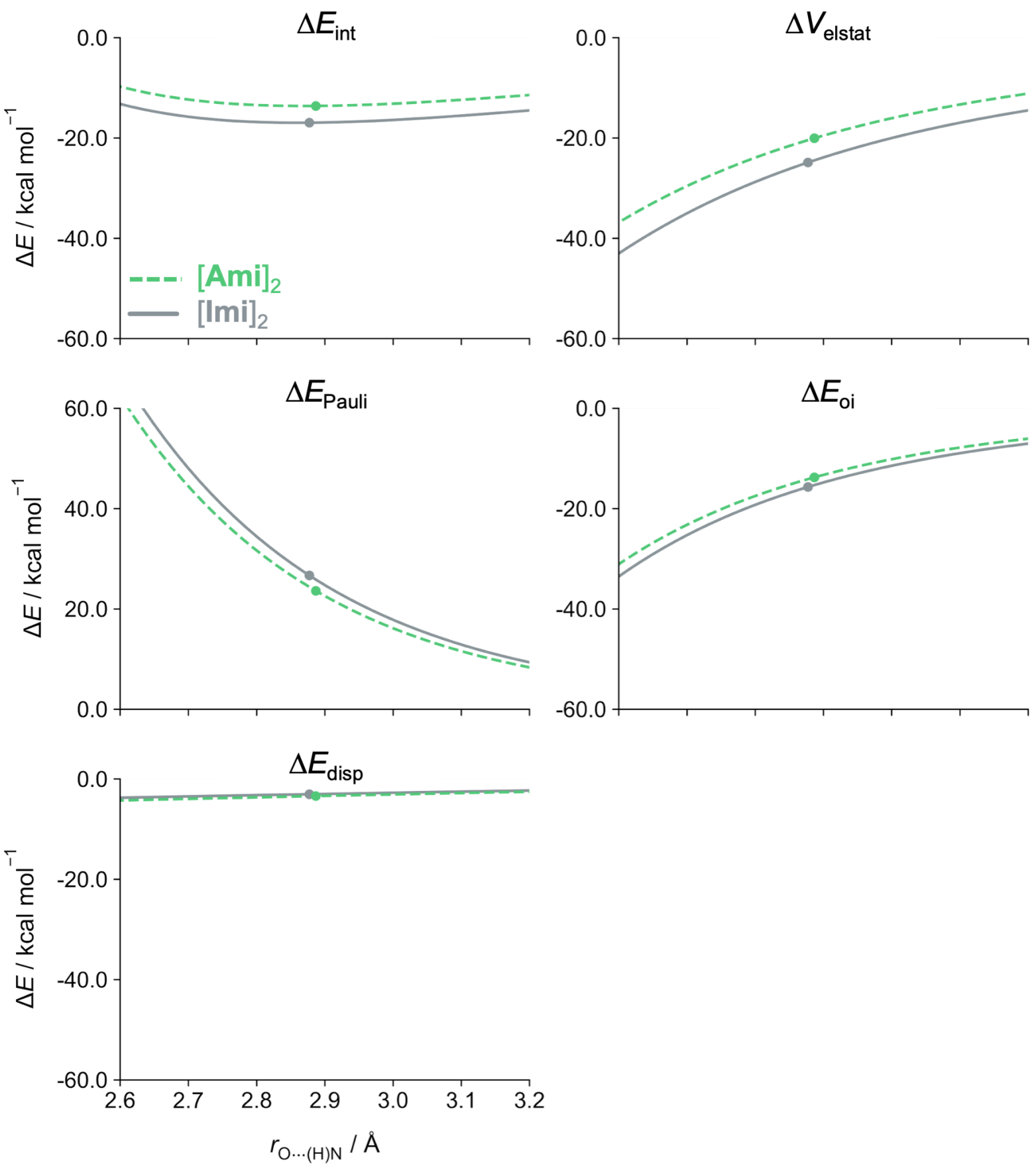

Figure S3. Energy decomposition analysis terms (in kcal mol^{-1}) including dispersion as a function of the hydrogen bond distance $r_{\text{O}\cdots\text{(H)N}}$ (in Å) for the simplified hydrogen-bonded complexes [**Ami**]$_2$ and [**Imi**]$_2$. The dots indicate the equilibrium $r_{\text{O}\cdots\text{(H)N}}$ distances.

S3 Fragmentation procedure and electron occupations

To quantify the strengths of primary and secondary interactions in [**Pyr**]$_2$, [**Ura**]$_2$, [**Ami**]$_2$, and [**Imi**]$_2$ dimers, the monomers were fragmented into hydrogen-bond acceptor and hydrogen-bond donor moieties. To account for the intrinsic charge polarization within each monomer, we assign partial charges to the moieties and adjust the orbital occupations accordingly. Since the monomers are planar and contain a π conjugated six-membered ring, we either remove or add electrons into the π system (the "AAA" irreducible representation in C$_s$ symmetry). In case of a positive partial charge, electrons are removed from the π HOMO with the number of electrons given by the magnitude of the charge. For example, the urea moiety in an **Ura** monomer has a partial positive charge of 0.073 electrons, thus 0.073 electrons are removed from the π irreducible representation (see Table S3). Oppositely, electrons are added to the π LUMO in case of a negative charge. Finally, to form the moieties, the planar systems needed to be broken, which would result in densities obtained from an open-shell state while the full monomer densities are obtained from a closed-shell states. We ensure closed-shell states of the moieties by terminating them with hydrogen atoms. Finally, the number of SCF cycles was set to 0 because the electrostatic interaction is calculated from unperturbed fragment densities.

Table S3. Specifications for the AMS input settings for each fragment considered in the pairwise fragmentations of [**Pyr**]$_2$, [**Ura**]$_2$, [**Ami**]$_2$, and [**Imi**]$_2$ dimers. Tabulated are the (partial) charge (in milli-electrons) computed through summing Voronoi deformation density (VDD) charges over atoms belonging to the associated fragment in the geometry of the interacting dimer, number of electrons N_{elec}, IrrepOccupations AMS input block (electrons), and the FragOccupations AMS input block with n_α // n_β electrons.

System	Fragment	Charge	N_{elec}	IrrepOccupations		FragOccupations	
				AA (σ)	AAA (π)	AA (σ)	AAA (π)
[**Pyr**]$_2$	C_3H_4O	–177	30	26	4.177	13 // 13	2.177 // 2
	C_2H_5N	177	24	20	3.823	10 // 10	1.823 // 2
[**Ura**]$_2$	C_3H_4O	–73	30	26	4.073	13 // 13	2.073 // 2
	CON_2H_4	73	32	26	5.927	13 // 13	2.927 // 3
[**Ami**]$_2$	CH_2O	–132	16	14	2.132	7 // 7	1.132 // 1
	NH_3	132	10	8	1.868	4 // 4	0.868 // 1
[**Imi**]$_2$	CH_2O	–57	16	14	2.057	7 // 7	1.057 // 1
	NH_3	90	10	8	1.910	4 // 4	0.910 // 1
	CH_2O^{SEI}	–33	16	14	2.033	7 // 7	1.033 // 1

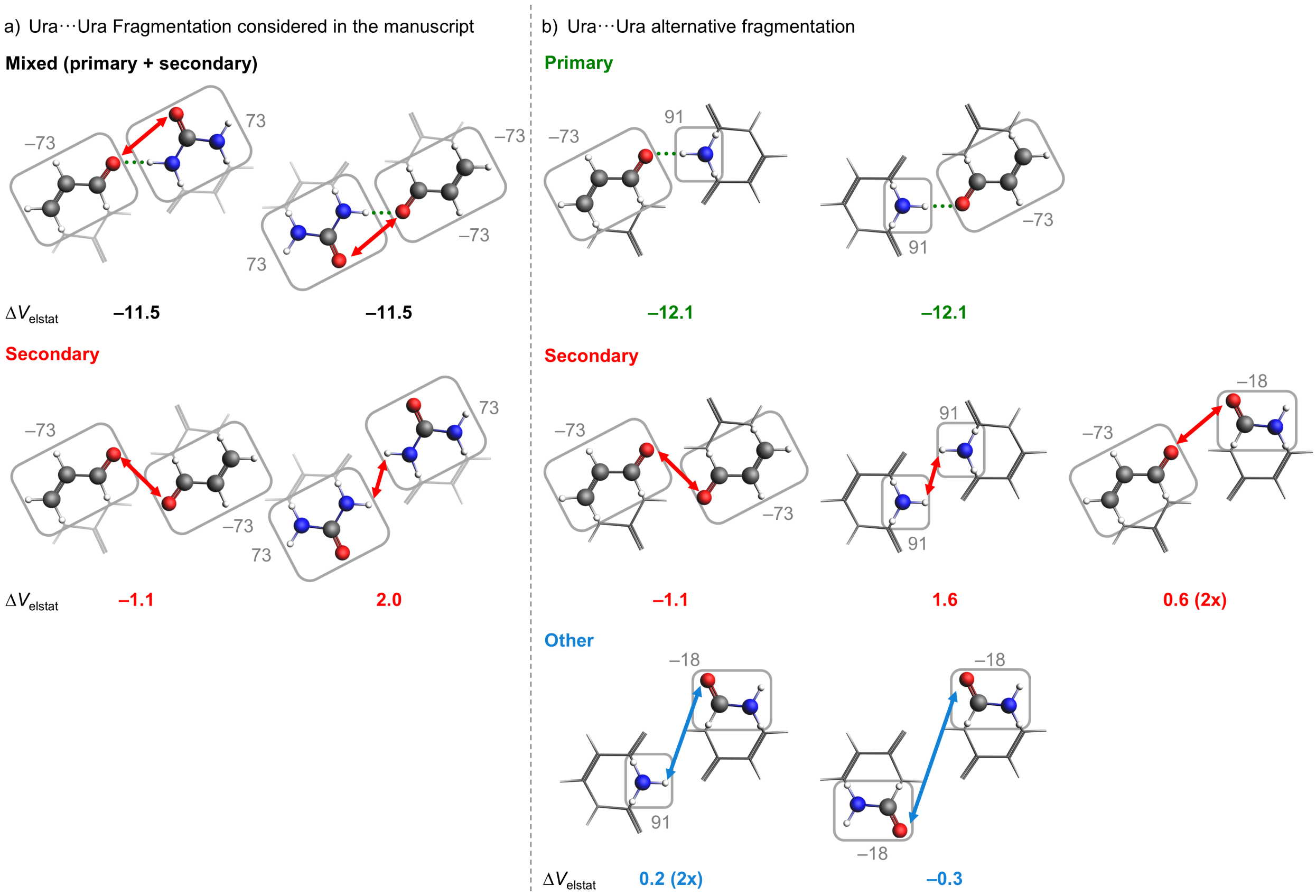

Figure S4. Comparison of the electrostatic interactions ΔV_{elstat} (in kcal mol^{-1}) between two **Ura•••Ura** monomers fragmented into smaller components. a) includes the mixed (in black) and secondary interactions (in red), and b), the primary (in green), secondary (in red), and other interactions (in blue). Partial charges are assigned to the hydrogen-bond acceptors and donor (grey values and boxes, in millielectrons). Calculated at consistent geometries with a $r_{\text{O}\cdots\text{(H)N}}$ distance of 2.780 Å at ZORA-BLYP-D3(BJ)/TZ2P.

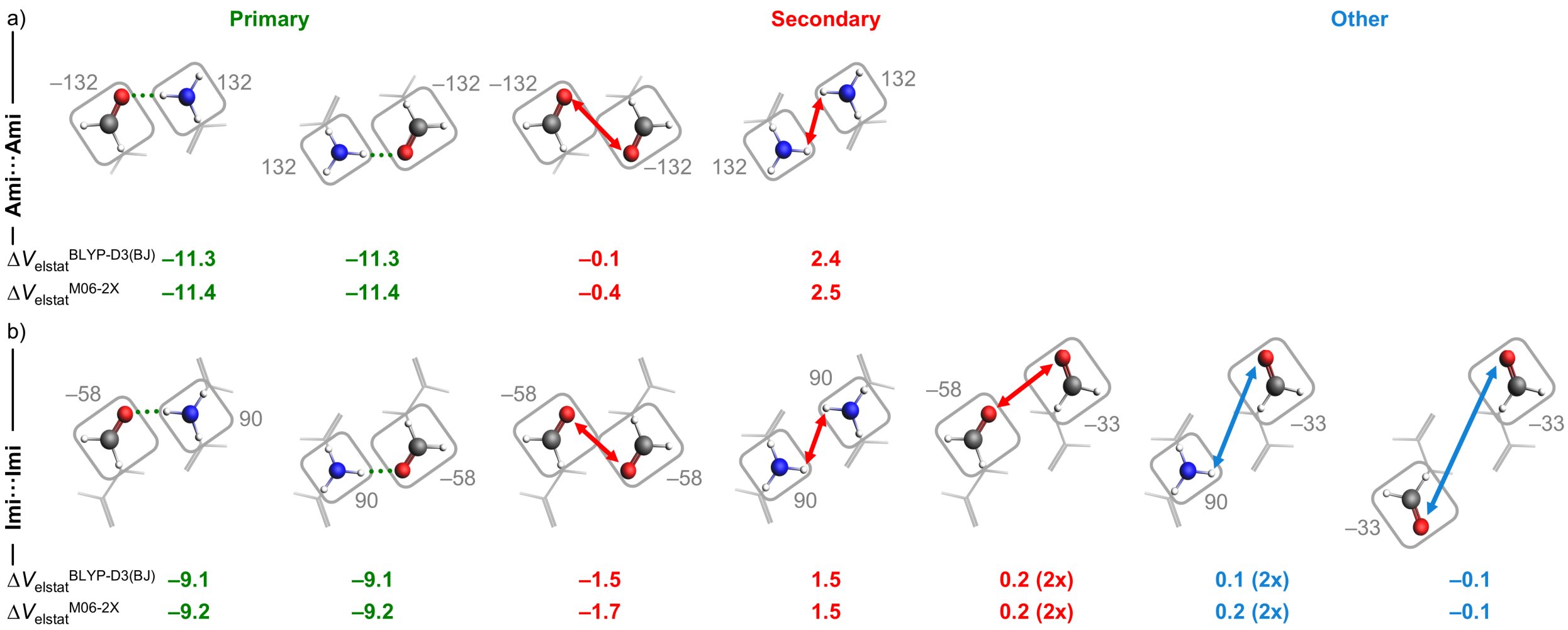

Figure S5. Electrostatic contributions ΔV_{elstat} (in kcal mol^{-1}) of a) fragmentations of the **Ami•••Ami** interaction into primary (in green) and secondary interactions (in red) between formaldehyde and ammonia, and b) fragmentations of the **Imi•••Imi** interaction into primary, secondary, and other interactions (in blue). Partial charges, as given in Figure 4 in the article, are assigned to the hydrogen-bond acceptor and donor (grey values and boxes, in millielectrons). Calculated at consistent geometries with a $r_{\text{O}\cdots\text{(H)N}}$ distance of 2.865 Å at a ZORA-BLYP-D3(BJ)/TZ2P (*i.e.*, $\Delta V_{\text{elstat}}^{\text{BLYP-D3(BJ)}}$) and ZORA-M06-2X/TZ2P//ZORA-BLYP-D3(BJ)/TZ2P (*i.e.*, $\Delta V_{\text{elstat}}^{\text{M06-2X}}$) level of theory.

The primary and secondary interaction components have also been quantified with neutral hydrogen-bond donor and hydrogen-bond acceptor fragments for which we consider the same fragments as above. **Figure S6** shows the electrostatic contributions obtained for [**Pyr**]$_2$ and [**Ura**]$_2$ and **Figure S7** shows electrostatic contributions of the primary and secondary interaction components for [**Ami**]$_2$, and [**Imi**]$_2$ dimers.

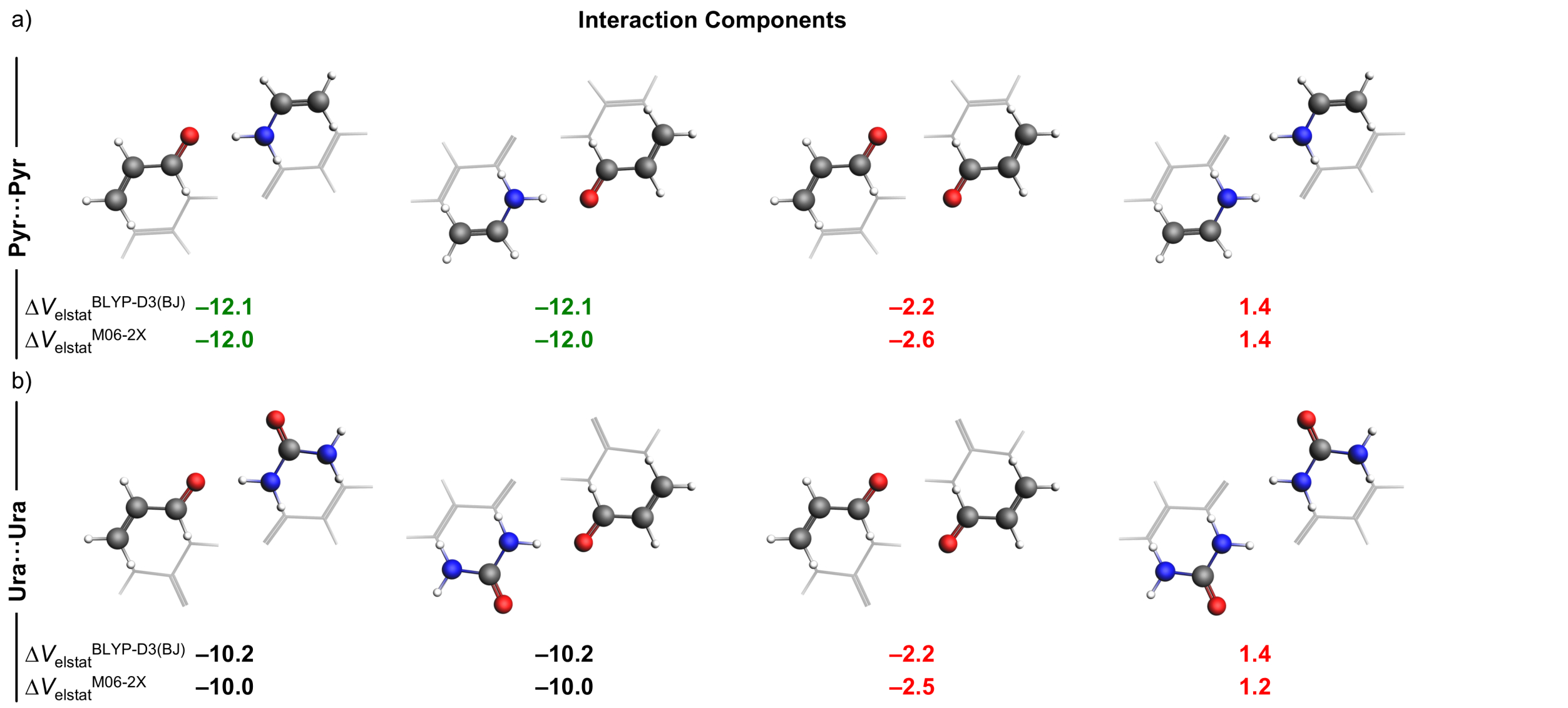

Figure S6. Electrostatic contributions ΔV_{elstat} (in kcal mol^{-1}) of a) fragmentations of the **Pyr•••Pyr** interaction into primary and secondary interactions between prop-2-al and aminoethylene, and b) fragmentations of the **Ura•••Ura** interaction into primary (in green) and secondary interactions (in red) between prop-2-al and urea. No partial charges are assigned to the fragments (*i.e.*, the fragments are neutral). Calculated at consistent geometries with a $r_{\text{O}\cdots\text{(H)N}}$ distance of 2.780 Å at a ZORA-BLYP-D3(BJ)/TZ2P (i.e., $\Delta V_{\text{elstat}}^{\text{BLYP-D3(BJ)}}$) and ZORA-M06-2X/TZ2P//ZORA-BLYP-D3(BJ)/TZ2P (i.e., $\Delta V_{\text{elstat}}^{\text{M06-2X}}$) level of theory.

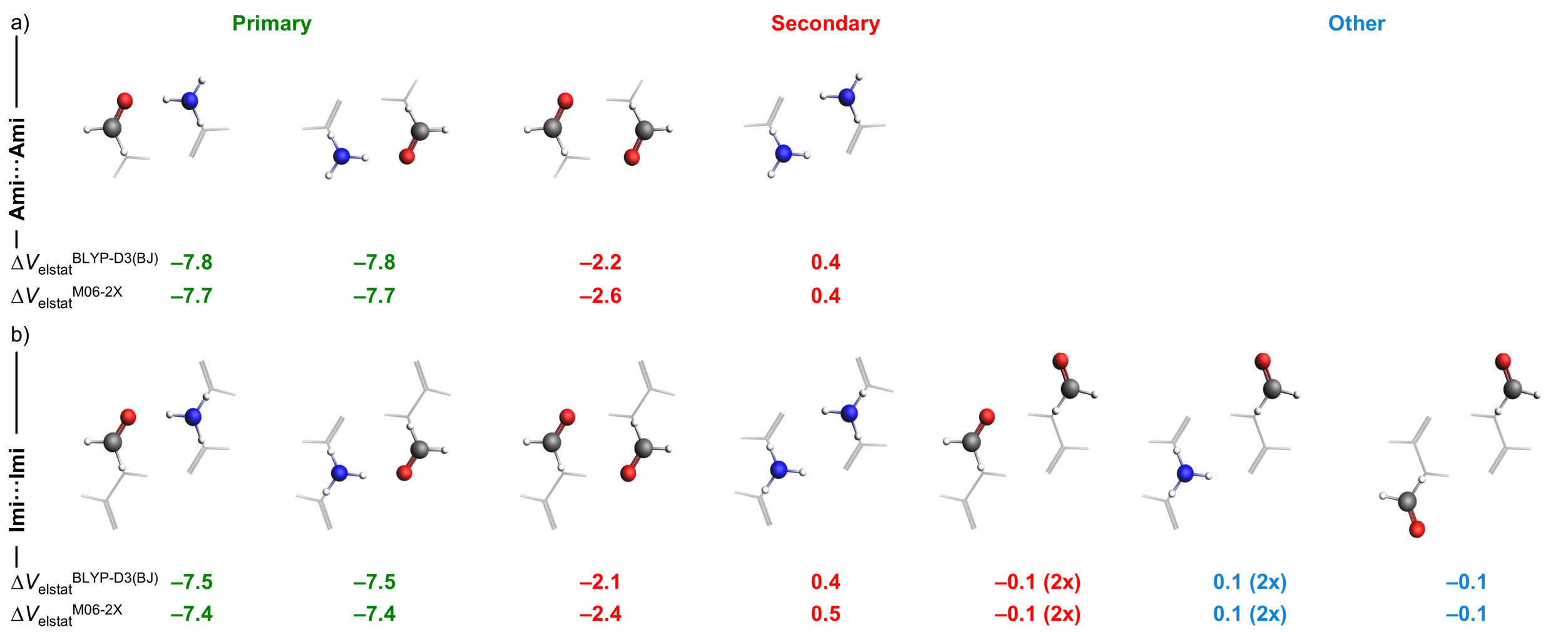

Figure S7. Electrostatic contributions ΔV_{elstat} (in kcal mol^{-1}) of a) fragmentations of the **Ami•••Ami** interaction into primary (in green) and secondary interactions (in red) between formaldehyde and ammonia, and b) fragmentations of the **Imi•••Imi** interaction into primary and secondary interactions. No partial charges are assigned to the fragments (*i.e.*, the fragments are neutral). Calculated at consistent geometries with a $r_{\text{O}\cdots\text{(H)N}}$ distance of 2.865 Å at a ZORA-BLYP-D3(BJ)/TZ2P (*i.e.*, $\Delta V_{\text{elstat}}^{\text{BLYP-D3(BJ)}}$) and ZORA-M06-2X/TZ2P//ZORA-BLYP-D3(BJ)/TZ2P (*i.e.*, $\Delta V_{\text{elstat}}^{\text{M06-2X}}$) level of theory.

S4 Orbital Interactions

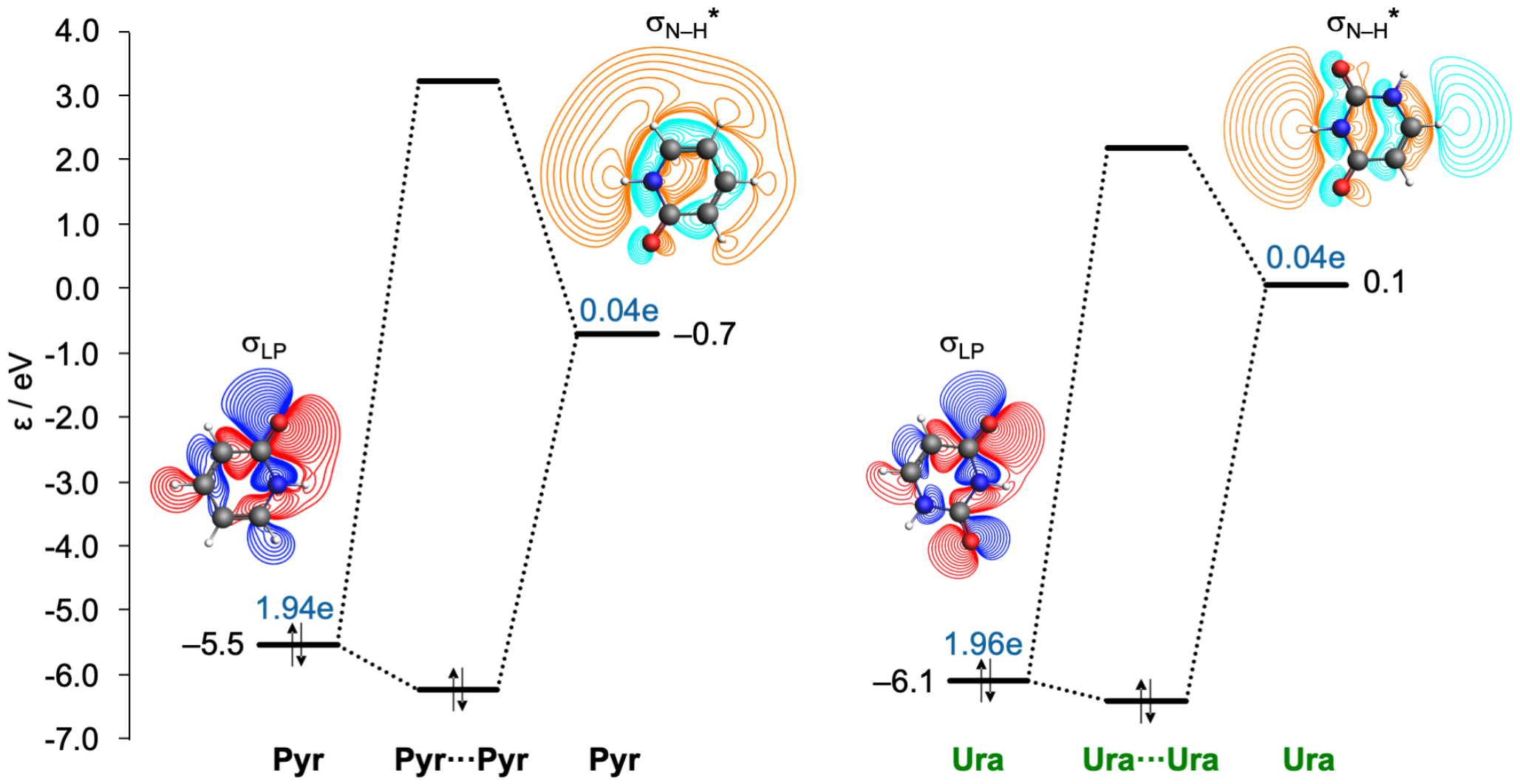

Figure S8. Molecular orbital diagram of the interaction between two monomers in [**Pyr**]$_2$ and [**Ura**]$_2$, computed at ZORA-BLYP-D3(BJ)/TZ2P.

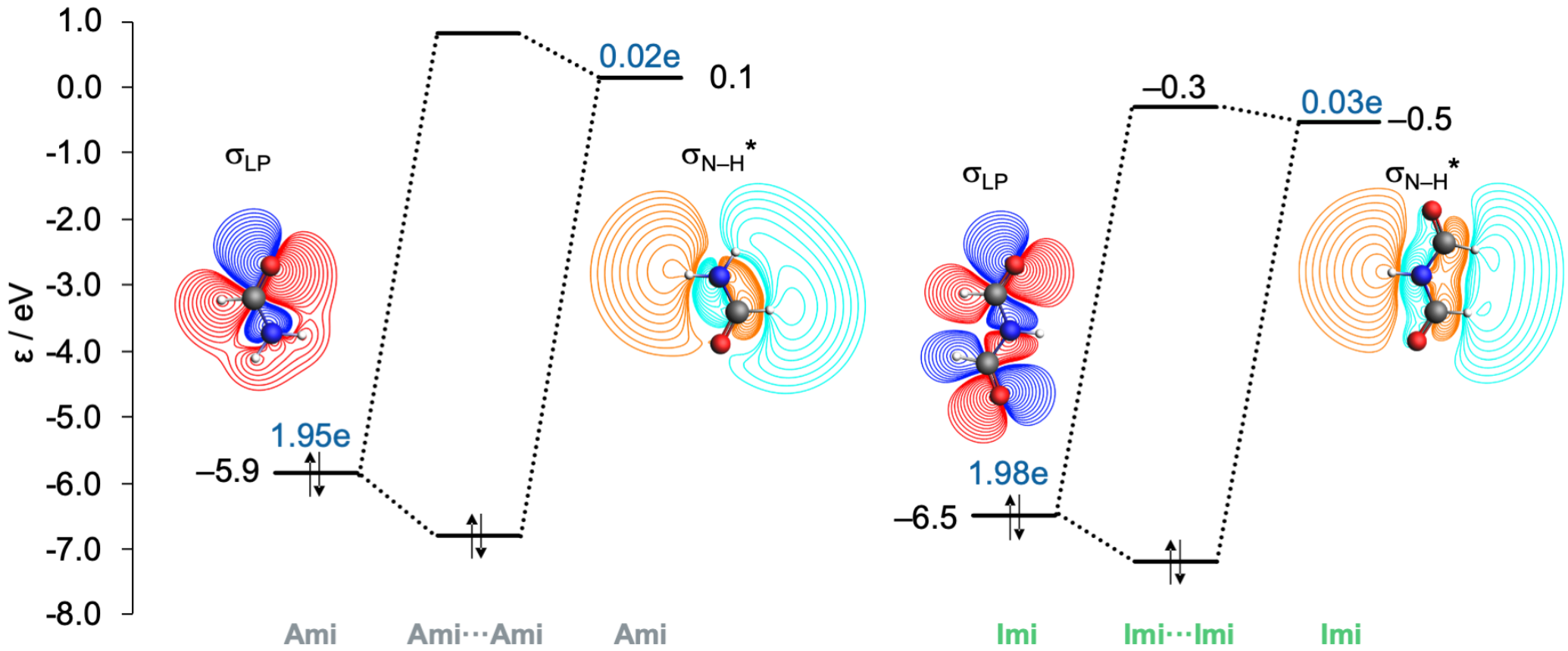

Figure S9. Molecular orbital diagram of the double-hydrogen interaction between two monomers in [**Ami**]$_2$ and [**Imi**]$_2$, computed at ZORA-BLYP-D3(BJ)/TZ2P.

S5 Cartesian coordinates

Table S4. Cartesian coordinates of 2-pyridone (**Pyr**), uracil (**Ura**), formamide (**Ami**), and formylformimidic acid (**Imi**) monomers and dimers, computed at a ZORA-BLYP-D3(BJ)/TZ2P level of theory with symmetry enforced.

2-Pyridone (Pyr) (C_s)
E = –1731.58 kcal mol^{-1}
H = –1670.81 kcal mol^{-1}
G = –1693.04 kcal mol^{-1}
N_{imag} = 0

```
C     -0.80230154     1.14421369     0.00000000
N      0.73526402    -1.11550306     0.00000000
C     -1.42680208    -0.13894187     0.00000000
C      0.56209530     1.27726020     0.00000000
C      1.44519511     0.12270502     0.00000000
C     -0.62562021    -1.24884767     0.00000000
H     -1.42774407     2.03541129     0.00000000
H      1.04488561     2.24993335     0.00000000
O      2.67955234     0.12280758     0.00000000
H      1.33169659    -1.93958738     0.00000000
H     -1.01039644    -2.26457600     0.00000000
H     -2.50582462    -0.24487515     0.00000000
```

2-Pyridone dimer [Pyr]₂ (C_{2h})
E = –3485.24 kcal mol^{-1}
H = –3362.40 kcal mol^{-1}
G = –3395.63 kcal mol^{-1}
N_{imag} = 0

```
C     -3.19784850     2.86159423     0.00000000
N     -1.70519493     0.56177384     0.00000000
C     -3.84769249     1.59854886     0.00000000
C     -1.82560796     2.95297814     0.00000000
C     -0.99874022     1.77125290     0.00000000
C     -3.06260232     0.47133525     0.00000000
H     -3.79711342     3.77043112     0.00000000
H     -1.31179314     3.90956746     0.00000000
O      0.26033188     1.77123733     0.00000000
H     -1.13512841    -0.32413246     0.00000000
H     -3.46391366    -0.53814377     0.00000000
H     -4.92884060     1.51413875     0.00000000
C      3.19782097    -2.86161271     0.00000000
N      1.70522112    -0.56175846     0.00000000
C      3.84769431    -1.59858050     0.00000000
C      1.82557965    -2.95296513     0.00000000
C      0.99874003    -1.77121821     0.00000000
C      3.06263247    -0.47134896     0.00000000
H      3.79706831    -3.77046110     0.00000000
H      1.31173784    -3.90953985     0.00000000
O     -0.26033257    -1.77117436     0.00000000
H      1.13517309     0.32415386     0.00000000
```

H	3.46396399	0.53812167	0.00000000
H	4.92884456	-1.51419791	0.00000000

Formamide (Ami) (C_s)
E = –783.11 kcal mol^{-1}
H = –752.70 kcal mol^{-1}
G = –771.09 kcal mol^{-1}
N_{imag} = 0

N	-0.36995426	-0.59376692	0.00000000
C	0.35661438	0.56533140	0.00000000
O	1.57769842	0.62611832	0.00000000
H	0.11196462	-1.48658785	0.00000000
H	-1.38201213	-0.57840222	0.00000000
H	-0.29431103	1.46730727	0.00000000

Formamide dimer [Ami]$_2$ (C_{2h})
E = –1581.77 kcal mol^{-1}
H = –1519.11 kcal mol^{-1}
G = –1544.77 kcal mol^{-1}
N_{imag} = 0

N	-1.71165113	0.60773969	0.00000000
C	-1.00627034	1.75657714	0.00000000
O	0.23057560	1.83891861	0.00000000
H	-1.23177110	-0.31110341	0.00000000
N	1.71164843	-0.60773806	0.00000000
C	1.00627272	-1.75657863	0.00000000
O	-0.23057285	-1.83892581	0.00000000
H	1.23176472	0.31110367	0.00000000
H	-2.72333619	0.64421150	0.00000000
H	-1.63689182	2.66880137	0.00000000
H	2.72333366	-0.64420597	0.00000000
H	1.63689832	-2.66880011	0.00000000

Uracil (Ura) (C_s)
E = –1797.48 kcal mol^{-1}
H = –1740.33 kcal mol^{-1}
G = –1764.12 kcal mol^{-1}
N_{imag} = 0

C	-0.76560024	1.15649661	0.00000000
N	0.73293124	-1.10058142	0.00000000
N	-1.36056060	-0.08743136	0.00000000
C	0.58053920	1.30531346	0.00000000
C	1.44937131	0.13156337	0.00000000
C	-0.64414270	-1.29595999	0.00000000
H	-1.45048801	1.99967731	0.00000000
H	1.04518554	2.28355951	0.00000000
O	2.67590221	0.13167708	0.00000000

H	1.29806631	-1.94741833	0.00000000
O	-1.19162433	-2.38958003	0.00000000
H	-2.36957993	-0.18731623	0.00000000

Uracil dimer [Ura]$_2$ (C_{2h})
E = –3608.74 kcal mol^{-1}
H = –3493.78 kcal mol^{-1}
G = –3528.73 kcal mol^{-1}
N_{imag} = 0

C	-3.26212876	2.84358375	0.00000000
N	-1.81432149	0.53464423	0.00000000
N	-3.88542231	1.61895312	0.00000000
C	-1.91055031	2.94669059	0.00000000
C	-1.09619537	1.74087506	0.00000000
C	-3.19708442	0.38735227	0.00000000
H	-3.92059938	3.70767446	0.00000000
H	-1.40781709	3.90569005	0.00000000
O	0.14580152	1.74084176	0.00000000
H	-1.25138528	-0.34253461	0.00000000
O	-3.78763095	-0.68221690	0.00000000
H	-4.89650955	1.54127412	0.00000000
C	3.26205936	-2.84362002	0.00000000
N	1.81439583	-0.53460215	0.00000000
N	3.88542634	-1.61904179	0.00000000
C	1.91046829	-2.94665150	0.00000000
C	1.09619449	-1.74079824	0.00000000
C	3.19715787	-0.38738204	0.00000000
H	3.92045853	-3.70776641	0.00000000
H	1.40769197	-3.90562607	0.00000000
O	-0.14580264	-1.74066153	0.00000000
H	1.25149536	0.34258528	0.00000000
O	3.78778057	0.68214138	0.00000000
H	4.89651743	-1.54140483	0.00000000

Formylformimidic acid (Imi) (C_{2v})
E = –1126.04 kcal mol^{-1}
H = –1088.60 kcal mol^{-1}
G = –1109.92 kcal mol^{-1}
N_{imag} = 0

N	0.00000000	0.00000000	0.48620704
H	0.00000000	-1.13861918	-1.26304310
H	0.00000000	1.13861918	-1.26304310
C	0.00000000	1.23620086	-0.15874133
C	0.00000000	-1.23620086	-0.15874133
O	0.00000000	2.30258315	0.42486166
H	0.00000000	0.00000000	1.50763850
O	0.00000000	-2.30258315	0.42486166

Formylformimidic acid dimer [Imi]₂ (C_{2h})
E = –2264.53 kcal mol^{-1}
H = –2188.21 kcal mol^{-1}
G = –2220.25 kcal mol^{-1}
N_{imag} = 0

N	-1.83650803	0.49064802	0.00000000
H	-3.70811237	1.42727831	0.00000000
H	-1.80101785	2.57266677	0.00000000
C	-1.14791787	1.67969811	0.00000000
C	-3.23404306	0.42363924	0.00000000
O	0.07728328	1.77625674	0.00000000
H	-1.28683949	-0.39223509	0.00000000
O	-3.86201799	-0.61471926	0.00000000
N	1.83650423	-0.49065029	0.00000000
H	3.70811683	-1.42726311	0.00000000
H	1.80103414	-2.57267000	0.00000000
C	1.14792575	-1.67970734	0.00000000
C	3.23403800	-0.42362838	0.00000000
O	-0.07727446	-1.77627692	0.00000000
H	1.28682485	0.39222747	0.00000000
O	3.86200404	0.61473575	0.00000000

Table S5. Cartesian coordinates of 2-pyridone (**Pyr**), uracil (**Ura**), formamide (**Ami**), and formylformimidic acid (**Imi**) monomers and dimers, computed at a ZORA-BLYP-D3(BJ)/TZ2P level of theory with no symmetry enforced (i.e., C_1)

2-Pyridone (Pyr) (C_1)
E = –1731.58 kcal mol^{-1}
H = –1670.80 kcal mol^{-1}
G = –1693.03 kcal mol^{-1}
N_{imag} = 0

C	-0.77784556	1.15952825	0.05824016
N	0.71154765	-1.12953454	-0.05365778
C	-1.42579791	-0.11128982	0.09892463
C	0.58559462	1.26612699	-0.03633120
C	1.44383672	0.09469679	-0.10036602
C	-0.64841510	-1.23648817	0.04083123
H	-1.38423705	2.06266037	0.10369143
H	1.08614342	2.22928474	-0.06771329
O	2.67499849	0.07089727	-0.18605657
H	1.29032221	-1.96499540	-0.09685043
H	-1.05205569	-2.24456959	0.06547920
H	-2.50409182	-0.19631689	0.17380865

2-Pyridone dimer [Pyr]₂ (C_1)
E = –3485.23 kcal mol^{-1}
H = –3362.39 kcal mol^{-1}

G = –3395.63 kcal mol^{-1}
N_{imag} = 0

C	-3.17522113	2.88532627	0.08751824
N	-1.70032505	0.57446857	0.04598170
C	-3.83425315	1.62713895	0.10392389
C	-1.80282079	2.96651108	0.05108943
C	-0.98511604	1.77865823	0.02810097
C	-3.05788918	0.49411179	0.08210707
H	-3.76747878	3.79860199	0.10403428
H	-1.28206158	3.91924825	0.03822377
O	0.27346651	1.76924621	-0.00554015
H	-1.13707905	-0.31564834	0.03005960
H	-3.46657785	-0.51235774	0.09188730
H	-4.91563013	1.55077810	0.13254405
C	3.17519345	-2.88534454	-0.08751778
N	1.70035137	-0.57445339	-0.04598224
C	3.83425471	-1.62717059	-0.10392416
C	1.80279257	-2.96649788	-0.05108873
C	0.98511613	-1.77862354	-0.02810081
C	3.05791924	-0.49412572	-0.08210775
H	3.76743344	-3.79863162	-0.10403348
H	1.28200649	-3.91922025	-0.03822230
O	-0.27346670	-1.76918323	0.00554054
H	1.13712389	0.31566941	-0.03006053
H	3.46662802	0.51233528	-0.09188841
H	4.91563362	-1.55083727	-0.13254447

Formamide (Ami) (C_1)
E = –783.11 kcal mol^{-1}
H = –752.70 kcal mol^{-1}
G = –771.09 kcal mol^{-1}
N_{imag} = 0

N	-0.38370981	-0.58407439	0.03235851
C	0.36965458	0.55599748	-0.03154299
O	1.58675360	0.58549728	-0.14400359
H	0.07278175	-1.48899436	-0.01408121
H	-1.39066070	-0.54270419	0.12629244
H	-0.25481941	1.47427819	0.03097684

Formamide dimer [Ami]$_2$ (C_1)
E = –1581.77 kcal mol^{-1}
H = –1519.11 kcal mol^{-1}
G = –1544.77 kcal mol^{-1}
N_{imag} = 0

N	-1.70119386	0.63051448	0.08313138
C	-0.98140605	1.76989939	0.05103049
O	0.25504262	1.83587022	-0.00788643
H	-1.23411175	-0.29460662	0.05854831

N	1.70119036	-0.63051266	-0.08314564
C	0.98140489	-1.76990080	-0.05110668
O	-0.25504014	-1.83587770	0.00787961
H	1.23410493	0.29460700	-0.05855192
H	-2.71113219	0.68037263	0.13126907
H	-1.59906367	2.69033382	0.08252811
H	2.71113197	-0.68036709	-0.13121889
H	1.59907288	-2.69033268	-0.08247741

Uracil (Ura) (C_1)
E = –1797.48 kcal mol^{-1}
H = –1740.33 kcal mol^{-1}
G = –1764.12 kcal mol^{-1}
N_{imag} = 0

C	-0.74782586	1.16734657	0.04145113
N	0.71605197	-1.11099069	-0.03948611
N	-1.35987077	-0.06793333	0.06972250
C	0.59852896	1.29685430	-0.02762948
C	1.44918075	0.11078387	-0.07431863
C	-0.66189478	-1.28658365	0.03078362
H	-1.41959658	2.02025001	0.07814591
H	1.07663046	2.26834374	-0.04980830
O	2.67393624	0.09335349	-0.13736096
H	1.26820687	-1.96580964	-0.06977434
O	-1.22445753	-2.37223833	0.05695602
H	-2.36888972	-0.15337634	0.12131863

Uracil dimer [Ura]₂ (C_1)
E = –3608.74 kcal mol^{-1}
H = –3493.77 kcal mol^{-1}
G = –3528.73 kcal mol^{-1}
N_{imag} = 0

C	-3.19516388	2.90851463	0.24177214
N	-1.79820891	0.57103876	0.13234187
N	-3.84156202	1.69663641	0.28397055
C	-1.84525912	2.98455846	0.14481153
C	-1.05765690	1.76263332	0.08321805
C	-3.18007302	0.45151958	0.23188254
H	-3.83430559	3.78563134	0.29112877
H	-1.32454054	3.93329214	0.11073030
O	0.18084141	1.73763734	-0.00645083
H	-1.25469841	-0.31727143	0.08969148
O	-3.79062168	-0.60595701	0.27273212
H	-4.85137529	1.63922303	0.35679001
C	3.19509275	-2.90855237	-0.24176336
N	1.79828302	-0.57099744	-0.13235074
N	3.84156425	-1.69672284	-0.28396695
C	1.84517869	-2.98451774	-0.14480670

C	1.05765517	-1.76255345	-0.08322210
C	3.18014754	-0.45155399	-0.23188769
H	3.83416878	-3.78571806	-0.29111186
H	1.32441340	-3.93322526	-0.11072246
O	-0.18084092	-1.73746207	0.00644426
H	1.25481311	0.31732758	-0.08970527
O	3.79076712	0.60587819	-0.27274215
H	4.85138103	-1.63935911	-0.35678350

Formylformimidic acid (Imi) conformer 1 (C_1)
E = –1126.04 kcal mol^{-1}
H = –1088.60 kcal mol^{-1}
G = –1109.92 kcal mol^{-1}
N_{imag} = 0

N	0.24886729	-0.41703052	-0.01927532
H	-1.62179928	0.49878261	0.11675192
H	0.32828570	1.66873293	-0.01691779
C	0.97695408	0.77115935	-0.06668497
C	-1.13956795	-0.49870880	0.07915102
O	2.18863724	0.81774558	-0.15285289
H	0.77192171	-1.29342073	-0.05979536
O	-1.75329879	-1.54726041	0.11962338

Formylformimidic acid (Imi) conformer 2 (C_1)
E = –1125.36 kcal mol^{-1}
H = –1087.90 kcal mol^{-1}
G = –1109.28 kcal mol^{-1}
N_{imag} = 0

N	0.15659300	-0.44843700	0.00000000
H	0.67322000	1.57756700	0.00000000
H	-1.77781400	-1.21554400	0.00000000
C	-1.22774600	-0.25167800	0.00000000
C	1.12054000	0.56979900	0.00000000
O	-1.77442200	0.83411600	0.00000000
H	0.51485100	-1.40285700	0.00000000
O	2.31477800	0.33703400	0.00000000

Formylformimidic acid (Imi) conformer 3 (C_1)
E = –1120.06 kcal mol^{-1}
H = –1082.79 kcal mol^{-1}
G = –1104.31 kcal mol^{-1}
N_{imag} = 0

N	0.23704000	-0.36071500	0.00000000
H	-1.39776900	-1.63687900	0.00000000
H	2.05712700	0.63360300	0.00000000
C	0.96470200	0.84508600	0.00000000
C	-1.15843300	-0.55022700	0.00000000

O	0.49873700	1.96064200	0.00000000
H	0.79625600	-1.21157100	0.00000000
O	-1.99766100	0.32006200	0.00000000

Formylformimidic acid dimer [Imi]$_2$ (C_1)
E = –2264.53 kcal mol$^{–1}$
H = –2188.20 kcal mol$^{–1}$
G = –2220.26 kcal mol$^{–1}$
N_{imag} = 0

N	-1.82635034	0.51820979	0.09960351
H	-3.68116450	1.48242804	0.20042012
H	-1.75961443	2.59947649	0.10138688
C	-1.12099637	1.69685548	0.06462167
C	-3.22274480	0.47190771	0.17401276
O	0.10376839	1.77513256	-0.00073120
H	-1.29075505	-0.37272829	0.06858613
O	-3.86519157	-0.55704258	0.20643742
N	1.82634051	-0.51821178	-0.09971595
H	3.68117710	-1.48241282	-0.20027138
H	1.75963509	-2.59948030	-0.10131294
C	1.12098771	-1.69686434	-0.06493316
C	3.22274514	-0.47189666	-0.17391335
O	-0.10376690	-1.77515327	0.00059584
H	1.29073473	0.37272088	-0.06868229
O	3.86519529	0.55705907	-0.20610408
